# Dock7 regulates AKT and mTOR/S6K activity required for the transformed phenotypes and survival of cancer cells

**DOI:** 10.1101/2023.01.03.522657

**Published:** 2023-01-03

**Authors:** Oriana Y. Teran, Matthew R. Zanotelli, Miao-chong Joy Lin, Richard A. Cerione, Kristin F. Wilson

**Affiliations:** 1 Department of Molecular Medicine, Cornell University, Ithaca, NY 14853, USA; 2 The Robert Larner, M.D. College of Medicine at The University of Vermont, VT 05405, USA; 3 Department of Chemistry, Cornell University, Ithaca, NY 14853, USA

## Abstract

Cancer cells, both within a developing tumor and during metastatic spread, encounter many stresses that require adaptive mechanisms to survive and maintain malignant progression. Here we describe a signaling complex involving the small GTPase Cdc42 and Dock7, a Cdc42/Rac GEF and unique Cdc42-effector, that has a previously unappreciated role in regulating AKT, mTOR, and other mTOR signaling and regulatory partners including the TSC1/TCS2 complex and S6K during serum starvation. Dock7 is highly expressed in triple-negative breast cancers and is essential for the malignant properties in nutrient-deprived growth conditions of several cancer cell lines. We find that Dock7 interacts with phosphorylated AKT to maintain a low, but critical activation of a rapamycin-sensitive and Raptor-independent mTORC1-like activity required for survival during nutrient stress. Following the knock-out of Dock7 from cancer cells, interactions between AKT and the phosphatase PHLPP increased while phosphorylation of AKT at Ser473 decreased, suggesting Dock7 protects AKT from dephosphorylation. The DHR1 domain of Dock7, previously of unknown function, is responsible for maintaining AKT Ser473 phosphorylation during serum starvation through an interaction requiring its C2-like motif. Together, these findings indicate that Dock7 protects and maintains the phosphorylation of AKT to sustain a tonic mTOR/S6K activity in cancer cells necessary for their resistance to anoikis and to prevent them from undergoing apoptosis during stressful conditions.

## Introduction

Mechanistic target of rapamycin (mTOR) is a serine/threonine kinase and a member of the PIKK family that functions within two distinct complexes, mTORC1 and mTORC2. mTORC1 is identified as a rapamycin-sensitive, nutrient- and mitogen-sensing complex defined by its interaction with the accessory protein Raptor^1^. It has been classically studied in the context of growth factor and amino acid signaling and shown to be recruited to the lysosome by the interaction between Raptor and the heterodimeric small GTPase RagA/B-C/D complex, which is activated by the Ragulator-v-ATPase. When bound to the lysosome, mTORC1 interacts with its direct activator, the small GTPase Ras homolog enriched in brain (Rheb)^2,3^. Rheb stimulates mTORC1’s kinase activity to promote a signaling cascade that triggers anabolic processes, including protein and lipid synthesis, while inhibiting catabolic metabolism such as autophagy. However, the regulation of mTORC2 and its functional roles have been less extensively studied.

Unlike mTORC1, mTORC2 is not inhibited by acute rapamycin treatment nor does it require Rheb to stimulate its kinase activity^4^. This complex consists of mTOR, Rictor, mLST8, and mSin1 and has been shown to promote cell survival during stress conditions (e.g., acidosis, hypoxia, nutrient deprivation)^5,6^. mTORC2 functions downstream of the energy-sensing kinase, AMP-activated protein kinase (AMPK), which phosphorylates and activates the mTOR catalytic subunit within mTORC2 in mouse embryonic fibroblasts (MEFs) and hepatocytes. AKT (Protein Kinase B), a major substrate of mTORC2, is then phosphorylated within its hydrophobic motif at Ser473, activating its ability to phosphorylate and inhibit numerous pro-apoptotic proteins, such as Bad and Caspase-9, thereby blocking apoptosis^7–9^. AKT also acts upstream of mTORC1 through an inhibitory phosphorylation of tuberous sclerosis complex (TSC)2 at Thr1462, as part of the TSC1/TSC2 GTPase Activating Protein (GAP) complex for Rheb, disabling its GAP activity and enabling Rheb to activate mTORC1^10–12^. Interestingly, even though AKT is widely regarded as a master regulator of survival, the mechanisms used by this kinase in the absence of growth factors and other nutrients to remain active and avoid dephosphorylation by phosphatases, such as PP2A and PHLPP, have remained elusive.

As regulators of cell proliferation, growth, survival, and metabolism, it is not surprising that members of the AKT/mTOR pathway are exploited in the context of cancer^13,14^. In fact, this pathway is hyperactivated in most cancers through numerous mechanisms, such as the amplification of receptor tyrosine kinases that signal to phosphatidylinositol-3-kinase (PI3K) (e.g., HER2 and EGFR), amplification of AKT itself, gain of function mutations in the catalytic subunit of PI3K (*PIK3CA*), or deletions in PTEN, one of the main negative regulators of the pathway^15–17^. AKT and mTORC1 hyperactivity have also both been shown to exacerbate transformation by impairing the DNA damage response (DDR) and reducing the stability of the genome^18,19^. Upon oncogenic transformation, cancer cells proliferate rapidly, doubling their lipid, nucleotide, and protein content with each cell division. Anabolic processes are thus enhanced, and catabolic processes are altered to meet the biosynthetic demands of the hyperactive growth of cancer cells, and these requirements are often met by the activation of AKT and mTOR^20–23^.

Various small GTPases have been implicated in the regulation of the mTOR/AKT pathway (e.g., Rheb and Rags). Small GTPases are molecular switches that cycle between an active GTP-bound state and an inactive GDP-bound state. The activation-deactivation cycles of small GTPases are regulated by GAPs which enhance their intrinsic GTPase activity and deactivate their signaling capability, and by Guanine nucleotide Exchange Factors (GEFs) which catalyze the dissociation of GDP and allow for the more abundant GTP in cells to bind and induce their signaling-active states^24^. The Rho GTPases are a subclass of the Ras superfamily and are traditionally known for playing major roles in cytoskeletal remodeling^25^. Although there have been multiple indications that Rho GTPases and their GEFs can activate mTOR/AKT signaling, and that their deregulation leads to cellular transformation^26–28^, the mechanisms responsible for how these processes are connected have been poorly understood.

In this study, we have identified the Cdc42/Rac GEF and Cdc42-signaling effector, Dock7, as a novel binding partner for AKT, mTOR, and several mTOR-associated proteins including its direct activator, Rheb, and its negative regulator, TSC1/2, which collectively work together as part of a unique survival response. Using several cancer cell lines, we show Dock7 is essential for cancer cell survival and that Dock7 expression promotes mTOR signaling and an enhanced transformed phenotype. Dock7 belongs to the atypical Dock180 family of Rho GEFs, which have two evolutionarily conserved Dock Homology Regions (DHR), DHR1 and DHR2^29,30^. DHR1 contains a C2-like motif that has been shown to be necessary for phospholipid binding in other Dock proteins^31–33^, while DHR2 has a putative dimerization region^34^ and a well-studied GEF domain that activates either Cdc42, Rac or both GTPases. We further demonstrate that the GEF activity of Dock7 is dispensable and that the less-studied DHR1 domain is responsible for maintaining AKT activity, resulting in the activation of an mTOR/S6K activity that is essential for cancer cells to survive distinct cellular stresses.

## Results

### Dock7 is highly upregulated in triple-negative breast cancers and required for the malignant phenotype of multiple cancer cell lines.

**Figure 1.**
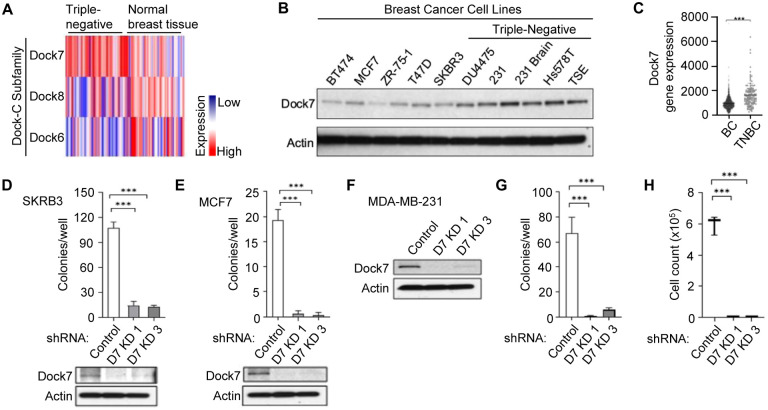
Dock7 is highly upregulated in aggressive triple-negative breast cancer cells and is indispensable for the transformed properties of breast cancer cells. (**A**) The Cancer Genome Atlas (TGCA) expression profile for Dock-C family members, Dock6, Dock7, and Dock8, in triple-negative breast cancer and normal tissue. (**B**) Protein expression of Dock7 in a breast cancer cell-line cohort. (**C**) Dock7 mRNA levels across patients with either receptor-positive (BC) or triple-negative breast cancers (TNBC). (**D** and **E**) Quantification of colonies formed in soft agar suspension for SKRB3 and MCF7, respectively. (Below) Western blot showing Dock7 protein expression after either control or Dock7 KD for each cell line. (**F**) Dock7 expression profile for MDA-MB-231 cells that were used for either (G) soft agar assay or (**H**) survival in serum-free media for 4 days. Soft agar assays were performed in triplicates, and colonies formed were quantified using ImageJ. The data shown in (**C**), (**D**-**E**), and (**G**-**H**) represent means ± SD; ***p < 0.001, and **p < 0.01

Due to the prominent role of aberrant Rho GEF activity in cancer^35^ and the largely unknown functions of the Dock-C atypical GEFs in oncogenic transformation, we used the cancer genome atlas (TCGA) Breast Cancer Dataset (BRCA) to examine the expression of the Dock-C subfamily in tumor tissue compared to normal breast tissue. In contrast with other members of the Dock-C subfamily, Dock7 is highly upregulated in triple-negative breast cancers compared to healthy mammary tissue (Fig. 1A). We then examined Dock7 protein expression in a panel of 10 different breast cancer cell lines (Fig. 1B), half of which were HER2^+^, ER^+^/PR^+^/HER2^+^, or ER^+^/PR^+^/HER2^−^ cell lines, while the remaining half were triple-negative breast cancer cell lines. Triple-negative breast cancer cells showed higher Dock7 protein expression compared to the other breast cancer cell lines, which corresponded to higher mRNA levels observed in triple-negative breast cancer patients (Fig. 1C).

**Figure 2.**
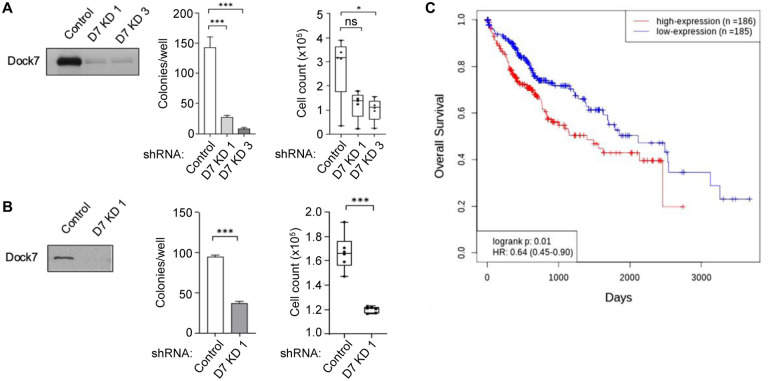
Dock7 is necessary for anchorage-independent growth and survival of both ovarian HeLa and lung A549 cancer cells, and its expression correlates with poor prognosis in liver cancer patients. (**A** and **B**) Soft agar colony formation assay and survival in serum-free media assays for HeLa cells and A549 cells, respectively, where Dock7 has been knocked down using shRNA. Western blot analysis shows Dock7 protein expression for each cell line. (**C**) Kaplan–Meier survival plot from The Protein Atlas showing a correlation between Dock7 mRNA expression and survival in liver cancer patients. Soft agar assays were performed in triplicates, and colonies formed were quantified using ImageJ. The data shown in (**A**-**B**) represent means ± SD; ***p < 0.001, and **p < 0.01, and not significant (n.s.)

To determine the impact of Dock7 on oncogenic transformation, we knocked down Dock7 in some of these breast cancer cell lines and assessed anchorage-independent growth and resistance to cell death, notable properties of transformed cells^36^. Following the Dock7 knock-down via short hairpin RNA (shRNA) in receptor-positive SKBR3 and MCF7 cells and triple-negative MDA-MB-231 breast cancer cells, anchorage-independent growth, as assessed by colony formation in soft agar, was dramatically decreased compared to control shRNA (Figs. 1 D, E, and G). Cancer cells exhibit a survival benefit over neighboring non-transformed cells in stress, nutrient-deprived microenvironments^36,37^, so we next studied MDA-MB-231 survival after four days in serum-free media. Relative to control cells, Dock7 knock-down cells had a significantly compromised ability to survive in the absence of nutrients (Fig. 1H). In HeLa cervical carcinoma cells and A549 lung carcinoma cells, Dock7 knock-down also resulted in a marked decrease in anchorage-independent growth and cell survival under serum-free conditions (Figs. 2A and B, respectively). Additionally, analysis of the human genome atlas indicated that higher levels of Dock7 mRNA expression is correlated with a poor prognosis for liver hepatocellular carcinoma patients (Fig. 2C). Together, these results reveal an essential role for Dock7 in supporting the malignant phenotypes of multiple cancer cell types during serum deprivation.

### Dock7 interacts with mTOR, its main negative regulator, the TSC complex, and stimulates mTOR activity.

**Figure 3.**
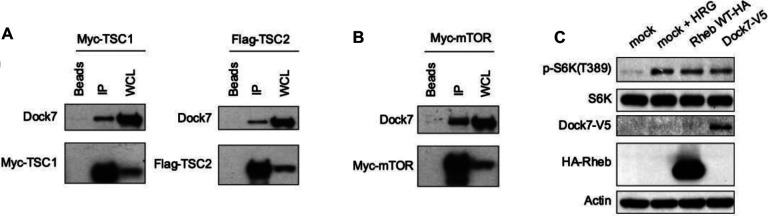
Dock7 interacts with mTOR and its main negative regulator and can stimulate mTORC1 signaling. (**A**) Either Myc-tagged TSC1, Flag-tagged TSC2, or (**B**) Myc-mTOR were transiently expressed in HEK293T cells and isolated with anti-Myc, or anti-Flag beads. Immunoprecipitated endogenous Dock7 protein was separated and analyzed by Western blot. (**C**) HeLa cells were transiently transfected with either WT Dock7 or Rheb for 48 h after which, the cells were starved overnight and collected for analysis. Heregulin treated (1 nmol/L, 30 mins) and mock-transfected cells were used as positive and negative controls, respectively. Western blots are representative of three separate biological replicates.

The mTOR pathway is highly activated in most cancers^13,14^, and proteomic studies have shown that Dock7 interacts with the main negative regulator of this signaling hub, namely the TSC complex (TCS1 and TSC2)^38^. To confirm an interaction between Dock7 and TSC1/2, we transiently transfected HEK-293T cells with either Myc-TSC1 or Flag-TSC2 and isolated tagged proteins by immunoprecipitation. We found that endogenous Dock7 co-immunoprecipitated with both TSC1 and TSC2 when cells are grown in complete media (Fig. 3A). Next, we examined whether Dock7 can interact with mTOR itself. Indeed, when Myc-mTOR was ectopically expressed and immunoprecipitated through its Myc tag, we observed the co-immunoprecipitation of endogenous Dock7 (Fig 3B). Consistent with these observations, we found that Dock7 co-migrated with mTOR, TSC1, and TSC2 as a high molecular weight species upon performing Blue Native-PAGE (BN-PAGE)^39^, a technique used to characterize the components of large metabolic complexes based on their native molecular mass (Supplemental Fig. S1A).

Given that TSC1/2 is a negative regulator of mTORC1, we investigated the ability of Dock7 to influence mTORC1 activity. HeLa cells were transiently transfected with full-length Dock7 and then cultured in serum-free media overnight to obtain a basal signal for S6K phosphorylation at Thr389, a well-known readout for mTORC1 activation^40–42^. Ectopic expression of Dock7-V5 stimulated mTOR activity, as indicated by a marked increase in p-S6K compared to control cells (Fig. 3C). The activation of mTOR by Dock7 was comparable to that obtained when cells were stimulated with Heregulin (an activator of ErbB2/HER2) or upon ectopic expression of the small GTPase Rheb, which directly activates mTORC1. These findings show a previously unidentified role for the Cdc42/Rac GEF Dock7 in mTOR regulation.

### Dock7 drives mTOR activity in a Cdc42-dependent, but GEF-independent, manner.

**Figure 4.**
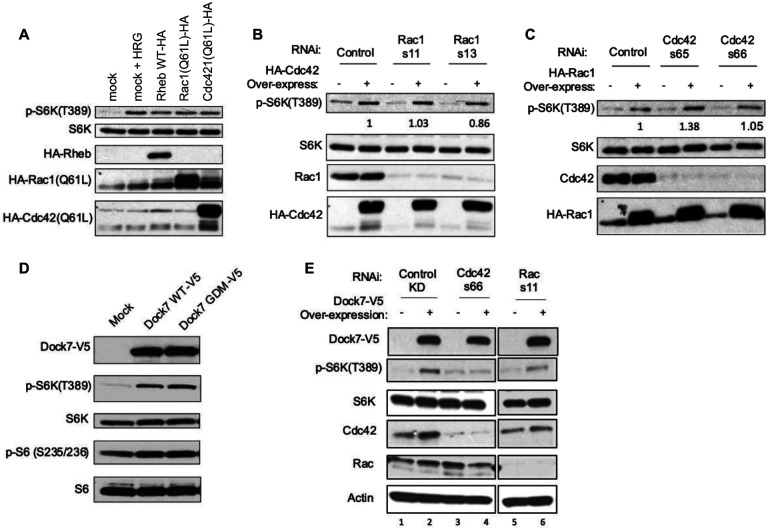
Dock7 stimulates mTOR activity in a Cdc42-dependent, but GEF-independent, manner. (**A**) Cells were either mock-transfected or transiently transfected with plasmids to overexpress HA-tagged WT Rheb or the active forms of Cdc42 and Rac. Heregulin treatment (1 nmol/L for 1 h) was used as a control to confirm mTORC1 signaling stimulation. (**B**) HA-tagged Cdc42 was overexpressed, and different Rac1 siRNAs were used to knock-down Rac protein. (**C**) HA-tagged Rac1 was overexpressed, and different Cdc42 siRNAs were used to knock-down Cdc42 protein. (**D**) Cells were either mock-transfected or transiently transfected with plasmids to overexpress either V5-tagged Dock7 WT or its GEF-defective mutant (GDM). (**E**) V5-tagged Dock7 was transiently overexpressed, while either Cdc42 or Rac was knocked down using siRNA. All experiments were performed in HeLa cells. After the genetic manipulation specified, cells were serum starved overnight (16–20 h) and collected for Western blot analysis. Western blots are representative of three separate biological replicates, and numbers under western blots represent fold difference between phosphorylation states when normalized to endogenous protein levels.

There have been various reports that Rac1 and Cdc42 are able to stimulate mTOR activity^26,28^; however, the mechanisms of activation have not been clearly elucidated. Given that Dock7 is both a Cdc42 and Rac GEF^43^, and because Dock7 associates with an mTOR-containing complex, we examined if Dock7 might function as a point of convergence for Cdc42/Rac1 and mTORC1 signaling. Thus, we first confirmed that Rac1 and Cdc42 were able to stimulate mTOR activity by overexpressing constitutively active forms of Rac1(Q61L) and Cdc42(Q61L) in HeLa cells followed by serum starvation overnight and Western blotting for S6K phosphorylation at Thr389 (p-S6K) to evaluate mTORC1/S6K activation. Both Cdc42 and Rac1 were indeed able to promote mTORC1 activity to a similar extent as Rheb (Fig. 4A). We then examined whether Cdc42 and Rac1 function independently of one another in their ability to signal to mTOR/S6K or if they are acting through a single signaling pathway. We overexpressed Cdc42(Q61L) while knocking down Rac1 and found that Cdc42 is effective at stimulating S6K in the absence of Rac1 (Fig. 4B). Similarly, Rac1(Q61L) is not dependent upon Cdc42 to activate S6K (Fig. 4C). However, the ability of both Cdc42 and Rac1 stimulate mTOR was significantly reduced in cells where Rheb was knocked down (Supplemental Fig. S2A and S2B). Taken together, these findings demonstrate the independent abilities of both Cdc42 and Rac1 to signal to mTORC1/S6K through Rheb.

GEFs activate small GTPases by stimulating GDP-GTP exchange, thus promoting their signaling-active states. Therefore, we tested whether the GEF activity of Dock7 was required to trigger mTORC1 signaling by creating a full-length Dock7 mutant construct that contains a mutation in the conserved, catalytic valine residue within the GEF domain that renders Dock proteins GEF-defective^44^. Interestingly, overexpression of the Dock7 GEF defective mutant (Dock7-GDM-V5) promoted S6K phosphorylation to a similar extent as wild-type Dock7, suggesting that Dock7 GEF activity is dispensable for the ability of Dock7 to activate mTORC1 (Fig. 4D). We have previously reported that the DHR2 domain of Dock7 is not only responsible for GEF activity but also contains an allosteric binding site for activated, GTP-bound Cdc42^45^. Using GST-tagged recombinant Cdc42 or Rac1 loaded with either GTPγS (i.e., a non-hydrolyzable form of GTP), GDP, or treated with EDTA to create a nucleotide-free state, we performed GST-pull-down assays using cell lysates from HEK-293T cells semi-stably expressing V5-tagged DHR2. We confirmed that V5-DHR2 bound best to GST-Cdc42, either when it was in a nucleotide-free state, as expected for a GEF, or when GST-Cdc42 was GTP-bound. However, this was not the case for Rac1, as the nucleotide-free state of GST-Rac was more effective than the GTP-bound form of the GTPase in pulling down the DHR2 domain (Supplemental Fig. S2C). Given that the GEF activity of Dock7 appeared dispensable in the activation of mTORC1, but that Dock7 contains an active Cdc42 binding site that is distinct from the GEF domain, we next probed whether Cdc42 could function as an upstream regulator of Dock7 to mediate its activity. To that end, we observed that the ability of transiently expressed Dock7 to activate mTORC1 activity was markedly reduced in cells when Cdc42 was knocked down (Fig. 4E). The role of Cdc42 in this activity was specific as the knock-down of Rac1 had no significant effect. Thus, these results suggest a Dock7 GEF-independent requirement for Cdc42 in the ability of Dock7 to regulate TORC1.

### Dock7 knock-out impairs transformative properties through decreased AKT activity and increased apoptosis.

**Figure 5.**
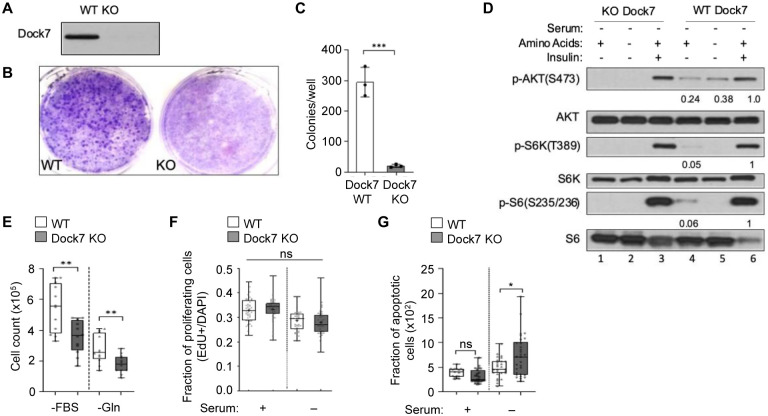
Dock7 knock-out impairs transformative properties through decreased AKT activity and increased apoptosis. CRISPR-Cas9 with Dock7 gene guiding sequence was expressed in cells and antibiotic resistance was applied. Once cells were selected, Dock7 protein expression was determined using western blotting (**A**) and then these cells were used as follows: (**B** and **C**) Cells were seeded and allowed to grow in full media or (**B**) in soft agar suspension for two weeks (**C**). Colonies formed in soft agar suspension were counted two weeks after seeding. (**D**) Cells were seeded and allowed to recover for a day before changing media to serum-free media for 24 h. Cells were then either collected (Lane 1 and 4), treated with 100 nM insulin for 1 h (Lane 3 and 6), or media was changed to HBBS to remove amino acids for 1 h (Lane 2 and 5) before being collected and used for Western blot analysis. (**E**) Cells were grown in either serum-free or glutamine-free media for two days before they were trypsinized and counted. (**F**) Cells were grown in either complete media or serum-free conditions for 20–24 h and then treated with EdU (10 μm) for 4 h. Cells were then fixed and EdU incorporation was determined. (**G**) Dock7 KO and WT HeLa cells were seeded, starved for 24 h, then stained with TUNEL and cleaved caspase 3 antibody to determine apoptotic index. DAPI stain was used to normalize for cell number. Western blots are representative of three separate biological replicates, and numbers under western blots represent fold difference between phosphorylation states when normalized to endogenous protein levels. Soft agar assays were performed in triplicates, and colonies formed were quantified using ImageJ. The data shown in (C and E-G) represent means ± SD; ***p < 0.001, and **p < 0.01, *p < 0.1, and not significant (n.s.)

To further study the role that Dock7 plays in survival and better control for residual effects imparted by partial knockdowns, we generated a Dock7 knock-out (KO) model system. Dock7 proved to be essential for the survival of MDA-MB-231 cells and we were unable to establish a complete KO of Dock7 using the Crispr-Cas9 system. However, we were able to create a Dock7 KO HeLa cell line (Fig. 5A) and observed a dramatic reduction in colony formation in the soft agar and focus formation assays following the complete genetic ablation of Dock7 expression (Figs. 5B and C, respectively), similar to knock-down experiments (Fig. 2A).

To better understand how the Dock7-mTOR signaling axis is regulated by stress, we then set out to identify signaling partners of Dock7 that may regulate mTORC1 activity in addition to TSC1/TSC2. Because Dock7 is essential when cells are challenged with significant stress as under conditions of anchorage-independent growth and nutrient deprivation, we initially focused on AKT, given its well-known role in promoting cell survival^7,8,19^. In wild-type HeLa cells, we consistently observed a low (basal) level of AKT activity which persisted upon the withdrawal of serum and was not significantly reduced further upon the additional removal of amino acids. In contrast, this basal AKT activity was absent in Dock7 KO cells under these conditions (Fig. 5D). However, growth factors (i.e., insulin; Fig. 5D, lane 3 and 6) and serum (Supplemental Fig. S3A) stimulated AKT phosphorylation and mTORC1 activity to the same extent in both the Dock7 KO and WT cells. Furthermore, during hypoxia in the presence of serum, we see a reduction in AKT phosphorylation when Dock7 is either knocked-down in MDA-MB231 cells or completely knocked out in HeLa cells (Supplemental Fig. S3B), again underscoring the importance of Dock7 function in responding to stress rather than to mitogenic signals. Finally, Dock7 was also required for survival in nutrient-starved conditions, as Dock7 KO significantly reduced survival relative to WT cells in serum-free as well as glutamine-deprived conditions (Fig. 5E). These data demonstrate that while Dock7 is not required for mitogenic growth, it imparts an essential survival function when cells are challenged with stress.

Despite the significant effects observed on the transformation potential of these cells, Dock7 KO did not impact proliferation in mitogenic growth conditions or under serum deprivation compared to control cells. (Fig. 5F). Since Dock7 was not essential for cell proliferation, we hypothesized Dock7 KO cells will undergo apoptosis at higher rates during nutrient deprivation. To examine this possibility, we identified apoptotic cells using a TUNEL assay for double-stranded DNA breaks in combination with immunofluorescence for cleaved caspase-3, as both markers indicate later, irreversible stages of apoptosis^46,47^. The extent of apoptosis during full serum conditions did not differ between WT and Dock7 KO cells; however, when serum was removed, Dock7 KO cells showed a significant increase in apoptosis (Fig. 5G). Western blotting confirmed increased levels of cleaved caspase-3 when Dock7 was knocked down in serum-starved HeLa cells (Supplemental Fig. S3C). Interestingly, Murine Embryonic Fibroblast (MEF) cells that contain reduced levels of Dock7 protein did not experience a decrease in survival when grown in serum-free conditions (Supplemental Fig. S3D). Taken together, these findings suggest that Dock7 plays a specific role in maintaining AKT activity to ensure the survival of cancer cells when they are exposed to challenging growth conditions.

### mTORC2 is required for Dock7-dependent AKT activity.

**Figure 6.**
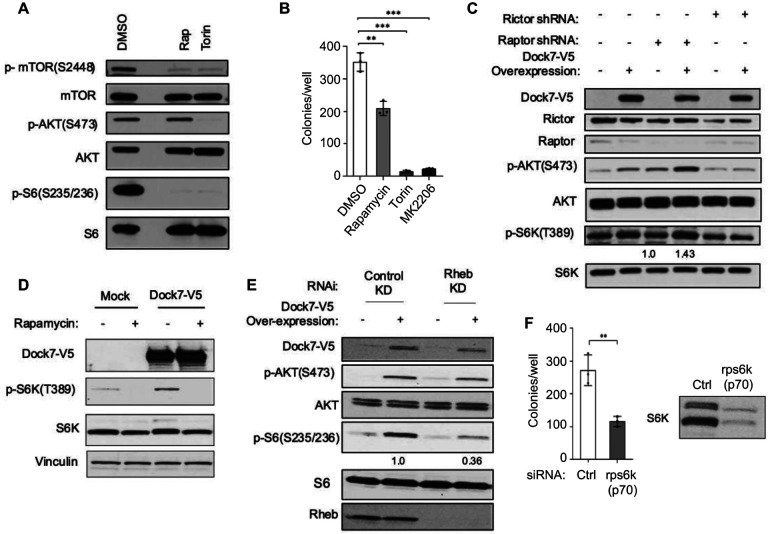
Dock7-mediated AKT phosphorylation in serum-deprived conditions requires Rictor, but not Raptor, and mTORC1-like activity is partially responsible for survival. (**A**) WT HeLa cells were seeded and culturedfor one day before media was changed to serum-free media containing either vehicle DMSO, Rapamycin (1 nM) or Torin (100 nM). Cells were treated for 20 h, then collected, and lysed for Western blot analysis. (**B**) WT HeLa cells were seeded in soft agar suspension and allowed to recover for a day before treatment began. Cells were then treated with 200 μl of complete media containing either vehicle DMSO, Rapamycin (1 nM), Torin (100 nM), or MK2206 (10 μM) on day 2, and every 3 days subsequently. Colonies were quantified 2 weeks after first drug treatment. (**C**) Either Rictor or Raptor was semi-stably knocked down with shRNA, and then full-length V5-tagged Dock7 protein was overexpressed. After 48 h, cells were starved overnight and collected for Western blot. (**D**) Dock7-V5 containing plasmid was transiently transfected in WT HeLa cells and cells were treated with serum-free media either containing vehicle control (DMSO, 1:100) or Rapamycin (1 nM). Cells were then collected and used for Western blot analysis. (**E**) V5-tagged Dock7 was transiently overexpressed in HeLa cells, and Rheb was knocked down using siRNA the next day. After 48 h, cells were starved overnight and collected for Western blot analysis. (**F**) S6 Kinase was knocked down using siRNA, and cells were seeded in soft agar suspension. Colonies formed were counted two weeks after. S6K protein levels were determined using western blotting. Western blots are representative of three separate biological replicates, and numbers under western blots represent fold difference between phosphorylation states normalized to endogenous protein levels. Soft agar assays were performed in triplicates, and colonies formed were quantified using ImageJ. The data shown in (**B** and **F**) represent means ± SD; ***p < 0.001, and **p < 0.01.

Given that mTORC2 is a classical regulator of AKT phosphorylation, we examined whether the stress-induced phosphorylation of AKT at Ser473 was dependent on mTORC2^48^. Following an overnight incubation in serum-free media, we observed a striking decrease in p-AKT at Ser473 when cells were treated with Torin, an ATP-competitive inhibitor of mTOR, but not upon treatment with Rapamycin, a mTORC1-specific inhibitor, whereas the p-S6 signal was downregulated by both inhibitors (Fig. 6A). The p-AKT Ser473 signal was moderately reduced when cells were treated with the PI3K inhibitor, LY924002 (Supplemental Fig. S4A). These observations confirm that mTORC2 activity is essential for the phosphorylation of AKT at Ser473 during Dock7-dependent signaling under serum deprivation.

Since mTOR exists in two distinct complexes, mTORC1 and mTORC2, each with different functions that are known to undergo crosstalk through feedback mechanisms^49,50^, we set out to determine whether mTORC1 and/or mTORC2 played a role in promoting the Dock7-dependent oncogenic transformation of cancer cells. To assess the role of AKT and each mTOR complex in Dock7 signaling, we performed soft agar colony formation assays where HeLa cells were seeded in suspension, allowed to recover for one day, and then treated with pharmacological inhibitors targeting mTORC1 (Rapamycin), AKT (MK2206), or both mTORC1/2 (Torin). Rapamycin inhibited soft agar colony formation by approximately 50%, while both MK2206 and Torin fully blocked anchorage-independent growth (Fig. 6B), suggesting mTORC1/2 and AKT may have an important role in the Dock7-mediated signaling needed to maintain malignant properties of these cancer cells.

To further explore the relevance of each mTOR complex, we semi-stably knocked-down Rictor or Raptor using shRNAs, while transiently expressing full-length Dock7. Following the knock-down of Rictor, Dock7 was no longer effective at elevating p-AKT Ser473 and p-S6K signals, indicating a necessary role for mTORC2. Unexpectedly, the knock-down of Raptor, the classical regulator of mTORC1, had no effect on the ability of Dock7 to stimulate S6K activity (Fig. 6C). Given that the Dock7-dependent phosphorylation of S6K appeared to involve an atypical mTORC1-like activity that was independent of Raptor, we next examined whether it was Rapamycin-sensitive in Dock7 overexpressing cells. Treatment with Rapamycin also blocked the ability of ectopically expressed full-length Dock7-V5 to promote the phosphorylation of S6K (Fig. 6D), comparable to what was found in wild-type cells. We also checked whether the direct activator of mTORC1, Rheb, was necessary for this Dock7-dependent activity since we observed the interaction between Dock7 and Rheb in pull-down experiments (Supplemental Fig. 4B). As seen in Fig. 6E, the knock-down of Rheb diminished the ability of Dock7 to stimulate mTORC1-like activity (e.g., p-S6), but AKT phosphorylation was not significantly affected. While p-S6K is routinely used as a readout for mTORC1 activity^51^, S6K activity has also been implicated in tumorigenesis^52–54^. Therefore, we examined its relevance to anchorage-independent colony formation. When we knocked down S6K using RNAi, there was a reduction in the ability of HeLa cells to grow in soft agar (Fig. 6F). These results were consistent with Rapamycin treatment (~50% reduction), suggesting that this mTORC1-like activity plays a significant role in the Dock7/AKT-dependent survival response. Collectively, Dock7 appears to regulate a distinct mTOR/S6K signaling activity downstream of mTORC2 and AKT as part of a stress-responsive signaling pathway.

### Dock7 protects AKT from dephosphorylation.

**Figure 7.**
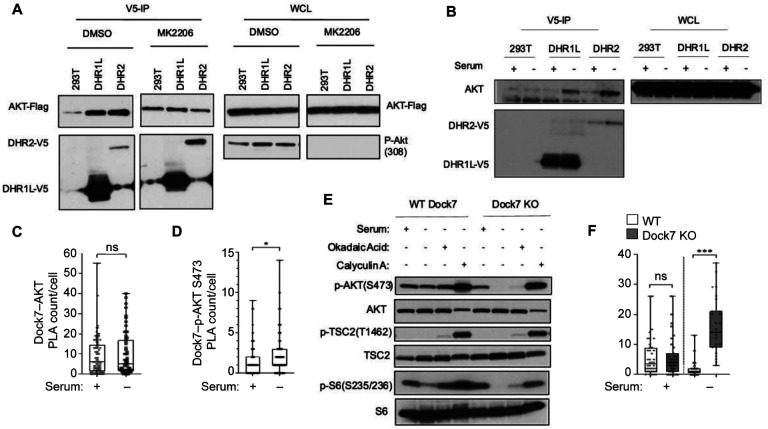
Dock7 and its domains interact and protect AKT from dephosphorylation. (**A**) Flag-tagged AKT was transiently overexpressed in HEK293T cells semi-stably expressing either V5-DHR1L or V5-DHR2. Cells were then either treated with vehicle control (DMSO, 1:1000) or with MK2206 (10 μM) for 16 h before being collected and lysed. Immunoprecipitation was performed using anti-V5 beads and complexes were resolved on an SDS-PAGE gel for Western analysis. (**B**) HEK293T cells semi-stably expressing either V5-DHR1L or V5-DHR2 were grown in full-serum or serum starved for 16 h, collected, lysed then used for immunoprecipitation and western blot analysis as described in (**A**). Proximity Ligation Assays (PLA) were performed to determine the number of direct interactions between endogenous Dock7 and either (**C**) endogenous AKT or (**D**) phospho-AKT in HeLa cells, respectively. (**E**) Cirspr-Cas9 Dock7 KO and WT HeLa cells were seeded and allowed to recover for 24 h. Media was then changed to either full serum media or serum-free media for 24 h. Serum-starved cells were then treated with either vehicle control DMSO (10 μl), Okadaic Acid (10 nM), or Calyculin A (50 nM) for 1 h before being collected for Western blot analysis. (**F**) Proximity ligation assay was performed on Cirspr-Cas9 Dock7 KO and WT HeLa cells to measure the number of complexes formed between AKT and PHLPP in the presence and absence of Dock7.

How AKT functions under stress-inducing conditions has not been thoroughly studied; however, it is evident that the levels of AKT activity necessary for cell survival are lower than, and not dependent upon, the canonical growth factor-stimulated pathways. Thus, we set out to explore the mechanistic basis by which Dock7, as a novel AKT regulator, maintains AKT activity during nutrient deprivation. We first examined Dock7 interactions with AKT by their ability to be co-immunoprecipitated from HEK-293T cells and saw that a small population of transiently overexpressed AKT can interact with Dock7 (Supplemental Fig. S5A). We then examined the abilities of the two evolutionarily conserved Dock7 domains, DHR1 and DHR2, to associate with AKT. For these studies, we used a full-length DHR2 limit domain, and a construct containing the DHR1 domain plus 183 amino acids of the C-terminal Dock7 linker region (DHR1_long_) that we hypothesized would express better than the DHR1 limit domain, DHR1_exact_ (Supplemental Fig. S5B) based on Alphafold predictions. Flag-tagged AKT was overexpressed in HEK-293T cells semi-stably expressing DHR1_long_-V5 or DHR2-V5, and co-immunoprecipitates were isolated using anti-V5 beads after cell lysis. To see if the DHR domains are preferentially bound to AKT when either in an activated or inactive state, cells were also treated with either the AKT inhibitor, MK2206, or vehicle control. Both DHR1_long_ and DHR2 were immunoprecipitated together with AKT, although these interactions were reduced when the cells were treated with MK2206 (Fig. 7A). We then examined whether the interactions between DHR1_long_ and/or DHR2 with AKT were enhanced when cells were stressed with serum deprivation. After overnight culture in serum-free media, we observed interactions between endogenous AKT and both DHR1_long_ and DHR2, whereas these interactions were not apparent in cells grown under normal conditions (full-serum) (Fig. 7B). We also used *in* situ proximity ligation assays (PLA) to detect and quantify endogenous Dock7 protein-protein interactions^55,56^. PLA showed Dock7 maintained an association with AKT during normal growth and serum-free conditions, and Dock7-pAKT Ser473 interactions increased under serum deprivation (Figs. 7C and D). Taken together, these results suggest that Dock7 associates with AKT, and during stress, Dock7 protects activated/phosphorylated AKT from de-phosphorylation/inactivation.

While it is possible that Dock7 acts as a scaffold to promote AKT activation by enhancing its interaction with mTORC2 during stress, we were particularly interested in exploring the ability of Dock7 to interact with activated/phosphorylated AKT and protect it from being inactivated by phosphatases upon serum withdrawal. To determine whether Dock7 blocked phosphatase interactions with AKT, we studied the impact of phosphatase activity on AKT following Dock7 knock-outs. Dock7 WT and KO HeLa cells were either cultured in full-serum or serum-free media overnight and treated with Okadaic Acid and Calyculin A, inhibitors of serine/threonine protein phosphatases^57^. Upon inhibition of phosphatase activity, phosphorylation of AKT at Ser473, as well as phosphorylation of its downstream effectors, TSC2 and S6, was maintained in Dock7 KO cells and AKT no longer required Dock7 for protection from dephosphorylation (Fig. 7E). We next examined the role of Dock7 in AKT interactions with PHLPP, which specifically dephosphorylates AKT at Ser473^58^. We used PLA to examine potential interactions between AKT and PHLPP in Dock7 KO or WT cells under serum-deprived conditions. A striking increase in the association of AKT and PHLPP was observed in Dock7 KO cells as compared to WT cells (Fig. 7F). These observations further indicate Dock7 interacts with active AKT to prevent its dephosphorylation by PHLPP and sustain a basal p-AKT activity during nutrient stress.

### Both the DHR1 and DHR2 domains rescue Dock7 knock-down and the DHR1 C2-like motif is necessary to protect phosphorylated AKT and sustain activity.

**Figure 8.**
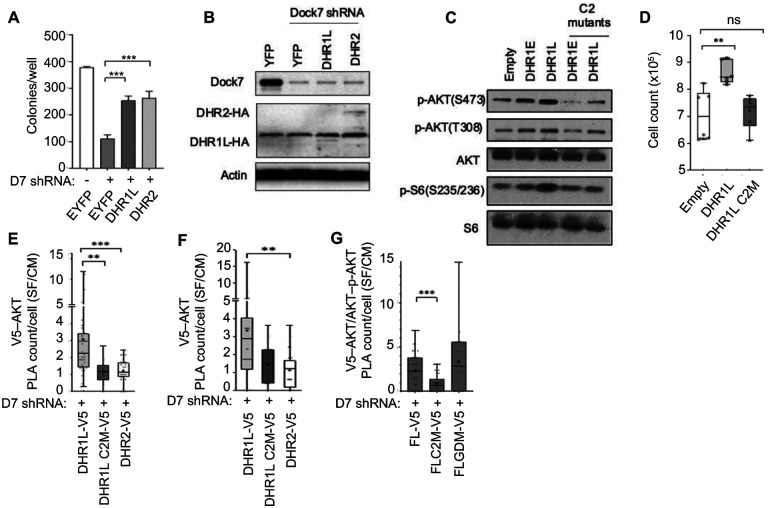
Both the DHR1 and DHR2 domains rescue Dock7 knock-down and the DHR1 C2-like motif is necessary to protect phosphorylated AKT and sustain activity. (**A** and **B**) Dock7 was semi-stably knocked down using shRNA and either an empty vector or vectors containing the limit DHR domains were expressed using lentiviral system. Cells were then seeded in either (**A**) soft agar suspension and counted two weeks later or (**B**) in 100 mm plates for protein analysis using western blotting. (**C**) Lentiviral transduction system was used to overexpress the specified domains and their mutants in HeLa WT cells. Cells expressing each plasmid were selected with antibiotics for 3–5 days, seeded, and allowed to recover for 24 h. Media was changed to serum-free media and cells were collected after 24 h for Western blot analysis. (**D**) Semi-stable HeLa cells overexpressing either an empty vector, DHR1L, or its C2 mutant were seeded, allowed to recover, and then allowed to grow for four days in serum-free conditions. Cells were then trypsin-treated and counted. (**E**-**G**) Dock7 was knocked down using shRNA in MDA-MB-231 cells, and then the specified constructs were overexpressed. Cells were then either grown in complete media or starved for 20–24 h. Proximity ligation assays (PLA) were used determine the number of interactions between endogenous AKT or phospho-AKT and each Dock7 construct. Western blots are representative of three separate biological replicates. Survival assays were performed in triplicates. Soft agar assays were performed in triplicates, and colonies formed were quantified using ImageJ. The data shown in (DG) represent means ± SD; ***p < 0.001, and **p < 0.01, *p < 0.1, and not significant (n.s.)

We attempted to rescue the effects resulting from the knock-down of Dock7 by expressing full-length Dock7 but were unable to achieve sufficient protein expression levels when using lentiviral transduction. Therefore, we tested the ability of the DHR1_long_ and DHR2 domains to restore the transformed phenotypes lost when Dock7 was knocked down. Initially, we examined whether DHR1_long_ and DHR2 could restore anchorage-independent growth in HeLa cells depleted of Dock7. Surprisingly, we found that soft-agar colony formation increased when either DHR1_long_ or DHR2 was expressed (Fig. 8A), despite their relatively low expression levels (Fig. 8B). We anticipated that the expression of the limit DHR2 domain, which functions as a Cdc42/Rac1 GEF, might be capable of rescuing the Dock7 KD. What was unexpected, however, was the ability of DHR1_long_ to rescue colony formation, since no activity has previously been described for this Dock7 domain. Our finding that both DHR1_long_ and DHR2 immunoprecipitated with AKT under serum deprivation (Figs. 7A and B) suggests the possibility that the DHR1 domain, by binding AKT and helping preserve its phosphorylated/activated state, might be responsible for restoring the transformation and survival of cancer cells after Dock7 knock-down.

DHR1 contains a putative C2-like motif, which has been shown to bind phospholipids and be important in the subcellular localization of Dock180 and Dock2^31,59^. Therefore, we examined the role of the C2-like motif within the DHR1 domain of Dock7. The sequences of all eleven Dock180-family proteins were aligned and two conserved positive amino acid residues on Dock7 were identified that might be capable of mediating interactions with a negatively charged binding partner. Site-directed mutagenesis was performed to create DHR1 C2 mutants (DHR1_exact_C2M and DHR1_long_C2M, Fig. S5B) by substituting alanine for the two conserved arginine residues, and then constructs expressing these mutants were introduced into HeLa cells. Antibiotic selection was applied to create semi-stable cell lines, and their ability to maintain cell survival and transformation was compared to wild-type DHR1 constructs. When either DHR1_exact_ or DHR1_long_ were ectopically expressed in these cells, the phosphorylation of AKT was elevated as was downstream signaling to S6K, compared to the vector control (Fig. 8C). In contrast, when the DHR1E and DHR1L C2 mutants were expressed in cells, the mutant DHR1 domains were unable to stimulate AKT and S6K activity (Fig. 8C). Interestingly, DHR1L showed a slightly greater potency to promote signaling compared to DHR1E. Therefore, we proceeded to further examine the DHR1L limit domain for further experiments and knocked down Dock7 then overexpressed V5-tagged DHR1L, DHR1L_C2M_, or DHR2 in MDA-MB-231 cells. We first performed survival assays and found that the decreased signaling observed in C2 mutants also reduced cell survival, as DHR1L C2M no longer promoted cell survival in serum free media (Fig. 8D). We then examined endogenous AKT interactions with limit domain constructs using the PLA multicolor kit for multiplex detection^60^. This allowed us to simultaneously detect V5-tagged Dock7 domain–AKT and AKT–p-AKT Ser473 interactions to identify the V5-AKT interactions that have AKT phosphorylated at Ser473. Compared to the DHR1L C2M and DHR2, DHR1L showed increased interactions with AKT and a higher amount of AKT interactions that were phosphorylated at Ser473 in serum-free media (Figs. 8E and F). We examined the C2 mutation in V5-tagged full-length Dock7 (FL C2M) and also found a decrease in interactions with AKT phosphorylated at Ser473 in serum-free media compared to wild-type full-length Dock7 (FL), confirming the critical function of the C2 domain in Dock7 interactions with p-AKT during serum deprivation (Fig. 8G). However, full-length GEF defective Dock7 (FLGDM) showed no change and had AKT interactions comparable to FL. Together, these results suggest that Dock7 interacts with AKT interacts through the C2-like motif within its DHR1 domain to maintain a basal level of AKT activity needed for survival.

## Discussion

The studies that we describe in this report highlight a new role for Dock7, a member of the Dock180 family of GEFs for Cdc42 and Rac, which has important consequences for cancer cell survival and tumorigenesis. The Dock180 family of atypical Rho GEFs consists of eleven members and is subdivided into four groups (A-D) based on sequence homology, and regulatory domains. The Dock-C subfamily members, which includes Dock7, lack the SH3 domain in Dock-A/B needed for interactions with ELMO proteins that drive Rac-dependent cytoskeletal remodeling^61–63^ and their biological function is largely uncharacterized^29^. Dock7 has mostly been studied in brain development where it regulates neuronal polarity and Schwann cell migration^64,65^. Recent studies have also examined the role of Dock7 in cancer, and these initial reports have suggested classical roles for Dock7-dependent activation of Cdc42/Rac in this context. For instance, in glioblastoma, Dock7 is involved in RAGE-dependent migration^66^, HGF-induced invasion^67^, and ligand-activated EGFR-mediated proliferation^68^. More recently, it was shown that Dock7 was essential for the survival of ovarian cancer cells after DNA damage and replication stress induced by chemotherapy. Gao, *et al*. showed Dock7 activated Cdc42 and Rac in the nucleus to ensure proper replication stress response through the stimulation of the serine/threonine protein kinase Pak1^69^. Here, we highlight an important new role for Dock7, where it potentiates AKT activity within a distinct mTOR signaling complex to maintain a malignant phenotype and cell survival during stresses like those observed within the tumor microenvironment.

The discovery of Cdc42-Dock7-AKT-mTOR as a stress signaling complex emerged from our efforts to understand how Cdc42 promotes the activation of mTOR in different cellular contexts. Early work by Blenis and colleagues showed that Cdc42 and Rac can stimulate S6K activity, a well-known downstream target of mTOR^26,28^. Studies from our laboratory then uncovered signaling connections between Cdc42 and mTOR that play important roles in cap-dependent mRNA splicing^70^, and the ability of multi-potent teratoma cells and embryonic stem cells to transition to neuro-progenitor/neural stem cells^71,72^. Proteomics studies showed that Dock7 interacts with the TSC1/TSC2 complex^38^, the GTPase-activating protein and negative regulator of the small GTPase Rheb. This provided a potential clue as to how Cdc42 may regulate mTOR, given that Dock7 serves as both a Cdc42-GEF and a binding partner for activated GTP-bound Cdc42^45^. However, it is still unclear how Cdc42 is initially activated to start this signaling process since we found that a GEF-defective mutation in Dock7 did not prevent it from signaling to mTOR. One possible explanation is that a pool of Cdc42-GTP pre-formed in growing cells is maintained in an active state during cellular stress by binding to the allosteric site on Dock7. It has been shown that signaling partners of GTP-bound Cdc42 can block GTP hydrolysis, helping Cdc42 remain in an activated state^73,74^. We found that like other Dock family members, Dock7 exists as a dimer (data not shown) and given that the allosteric binding site for Cdc42 sits proximal to the dimerization domain, it is attractive to envision that allosteric Cdc42 binding might regulate conformational changes within the Dock7 dimer. These changes could then allow Dock7 activation and the assembly of the Dock7/AKT/mTOR stress-response complex.

We then identified additional components that comprise the Cdc42-Dock7 signaling complex by showing that in addition to TSC1/TSC2, Dock7 can interact with Rheb, mTOR, and AKT. Moreover, we found that the ectopic expression of Dock7 gives rise to a Rapamycin-sensitive activation of mTOR/S6K in serum starved cells, due to its ability to preserve a basal level of AKT activity. Increasing evidence now suggests distinct low-level activity is critical in cancer. Ligand-activated EGFR signaling in glioblastomas promotes proliferation while inhibiting invasion to produce small hyperproliferating non-invasive tumors with increased survival^68^, and low expression of the metabolic enzyme PHGDH, which activates aberrant protein glycosylation to potentiate breast cancer dissemination and metastasis^75^. The Dock7-dependent low level of AKT signaling activity might have often been mistaken as simply background, but our work here demonstrates that Dock7 is critically important for cell survival. Inhibition of this basal AKT activity completely prevents cells from growing in soft agar, while the knock-down of S6K reduced the ability of cancer cells to grown in soft agar by half, suggesting that S6K activity is an important AKT signaling effector in the Dock7 survival response. Although collectively these observations point to the ability of Dock7 to regulate mTORC1, it is generally regarded that mTORC1 is inhibited when growth conditions are limited due to the phosphorylation of the mTORC1-defining subunit, Raptor, by AMPK. Our finding that the Dock7-dependent activation of S6K does not require Raptor, allows for an mTORC1-like activity that evades classical downregulation events during nutrient deprivation. Our study is not the first to report of a Rapamycin-sensitive, Raptor-independent mTORC1-like activity^76,77^ and in contrast to canonical mTORC1 signaling, Raptor-independent mTOR complexes may provide a mechanism to generate a very targeted and context-dependent mTOR/SK6 activity when canonical mTORC1 signaling is disabled.

Another unique aspect of this signaling complex is our discovery that the lesser studied DHR1 domain of Dock7 binds to phosphorylated AKT and helps to maintain a basal level of AKT activation necessary for ensuring cancer cell survival and blocking apoptosis when challenged by stresses, such as a nutrient deprivation. Other proteins have also been shown to protect AKT from dephosphorylation by phosphatases^78,79^ and Dock6, another Dock-C member of the Dock180 family, has been shown to interact with AKT and be reciprocally regulated by AKT and the phosphatase PP2A^80^. However, Dock7 binding to the phosphorylated AKT appears to occur through a distinct Dock7 DHR1-specific mechanism since C2 and C2-like motifs have not previously been implicated in binding to phospho-proteins. Very little is known about the DHR1 domains of the Dock family of atypical GEFs relative to the DHR2 domains, which confer the better-studied GEF activity. For two members of the family, Dock180 and Dock2, the DHR1 domain is known to be a membrane phospholipid motif that mediates the localization of these proteins^33,59^. We attempted using different lipid conjugated beads to pull-down Dock7 DHR1 and its C2 mutants from lysates, but these efforts failed to identify any lipid-binding preferences (unpublished data). As such, we were surprised to find that DHR1 rescued anchorage-independent growth and signaled to S6K in cells depleted of Dock7, suggesting the DHR1 domain provided an essential function for the Dock7 stress response. We found that the C2-like motif of the DHR1 domain of Dock7 bound phosphorylated AKT and protected it from dephosphorylation by its phosphatase, PHLPP, to preserve a pool of active AKT during serum deprivation. Structural determinations of either AKT phospho-peptides or recombinant AKT bound to DHR1 will be helpful in determining how this interaction occurs within the C2-like motif of DHR1. The finding that DHR1 promotes AKT activity by preserving its activated state explains how DHR1 can propagate signaling and give rise to transformed phenotypes, thus providing a new model for how the activity of AKT can be maintained during stress to promote survival.

Thus far, we have not definitively determined the cellular localization of the Cdc42-Dock7-AKT-mTOR signaling complex, either in growing cells or in cells that have been deprived of nutrients. Identifying where this signaling node assembles in cells will be important for several reasons, especially to understand how it relates to classical mTORC1. It has been well documented that mTORC1 is activated at the lysosome in response to mitogenic signaling and amino acids^1,81^. While we have not identified the cellular compartment(s) in which this Dock7 signaling node resides, immunofluorescence studies indicate that it does not colocalize with lysosomal markers (data not included). Additionally, this multi-protein complex does not appear to localize to the plasma membrane or with any other organelle marker we have examined to date. It will be important to elucidate where these Dock7/AKT/mTOR signaling complexes reside as the localization may be coupled to the specific functional outcomes of this signaling event and shed light on how they promote survival.

## Conclusions

**Figure 9.**
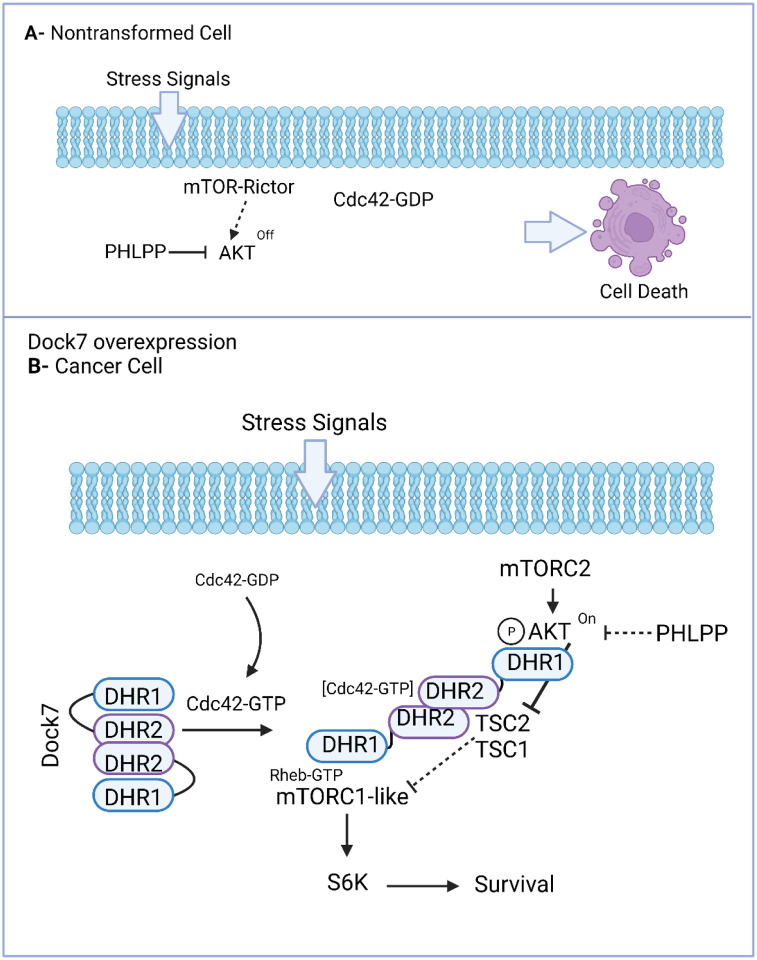
Proposed model of Dock7-mediated cellular response to stress. (**A**) In normal tissues, chronic stress will promote cell death. (**B**) When Dock7 is overexpressed in cancer cells and cells are challenged with stress, Dock7 will be able to maintain AKT phosphorylated to inhibit apoptosis and promote survival. Created with BioRender.com

Here we describe how the Dock7 member of the Dock180 family of GEFs is overexpressed in different cancers and plays an important role in oncogenic transformation. This role is distinct from those observed for other Rho GEFs that activate Cdc42 and/or Rac to contribute to transformed phenotypes through conventional mechanisms such as by promoting migration and invasion. Rather, we have discovered a stress-responsive survival signaling hub where Dock7 sequesters a population of active AKT to promote a Raptor-independent, mTOR/S6K activity, with both the AKT and mTOR/S6K activities playing a necessary role in cell survival. In Figure 9, we present a working model for Dock7 stress signaling, where an autoinhibited conformation of Dock7 responds to cellular stresses by undergoing a conformational change. Specifically, GTP-bound Cdc42, by interacting with an allosteric binding site distinct from the GEF domain on DHR2, induces an active/open conformation of Dock7 in response to stress signals. This would expose the C2-like motif within DHR1 to bind phosphorylated AKT and prevent its dephosphorylation by phosphatases, such as PHLPP. Other regions on Dock7 might serve as scaffolds both to enhance the interactions between active AKT and downstream effectors such as the TSC complex, and to assemble mTOR and its activator Rheb to generate a targeted, stress-dependent mTOR/S6K activity. As a first report of this Dock7 survival signaling hub, it will be necessary moving forward to better understand how the identified players are coming together spatiotemporally given the large molecular size of the complex, as well as elucidate the 3D structural features of the different protein-protein interactions that comprise this signaling node. Additionally, it will be important to perform proteomic and phospho-proteomic studies to define the global changes that occur downstream of the AKT-Dock7 signaling axis during stress, and to better distinguish the functions and molecular mechanisms of this distinct signaling node from the previously described growth-factor-dependent AKT/mTOR pathways.

## Materials and Methods

### Cell lines, cell culture, and reagents.

HeLa cervical carcinoma and breast cancer cell lines were obtained from American Type Cell Culture Collection (ATCC) and maintained at 37°C, 5% CO_2_ in RPMI 1640 (ThermoFisher) supplemented with 10% fetal bovine serum (FBS; ThermoFisher). TSE breast cancer cells were kindly supplied by Dr. Steven Abcouwer (University of Michigan) and the MDA-MB-231 cells metastasized to brain was kindly supplied by Dr. Joan Massagué (MSKCC,^82^). HEK-293T cells (ATCC) were maintained at 37°C, 5% CO_2_ in DMEM supplemented with 10% FBS (ThermoFisher). For growth factor stimulation, cells were seeded in 100 mm dishes (Corning) at 7×10^5^ cells/dish, serum-starved for 20–24 h, then stimulated with Heregulin β (HRG), EGF domain, residues 178–241 (Sigma-Aldrich) at the concentration and times indicated, followed by cell lysis. All cell lines were tested and found negative for mycoplasma contamination.

### DNA constructs, siRNA, and shRNA.

Rac1, Cdc42, Rheb, Dock7, wild-type and point mutation constructs used for transient transfections were cloned in our laboratory into pcDNA3.1 (Thermo Fisher). TSC1, TSC2, and mTOR DNA constructs were obtained from Addgene (plasmid #12133, #14129, #1861)^5,10,83^. Dock7 lentiviral constructs, Dock7 truncations, Rac1, Cdc42, Rheb, and YFP used for transformation assays were cloned into pSIN-EF2^84^. The Dock7 DHR1 construct contains amino acids (561–910), and the Dock7 DHR2 construct contains amino acids (1571–2130). GST-Rheb was cloned into pGEX (GE Healthcare Life Sciences). Silencer Select siRNAs of Rheb (s12019, s12020, s12021), Rac1 (s11711, s11713), and Cdc42 (s2765, s2766, s2767) were purchased from Thermo Fisher. Dock7 shRNAs and negative control were purchased from Sigma (shRNA1: TRCN0000377466, shRNA3: TRCN0000365143, shRNA6: TRCN0000365145) and virus particles were generated according to manufacturer’s protocol.

### Transfection.

Cells were seeded in 100 mm dishes (Corning) at 7×10^5^ cells/dishes then transfected with 4 μg DNA using Lipofectamine and Plus Reagent according to the manufacturer’s protocol (ThermoFisher). Cells recovered in complete medium for 3 h followed by serum starvation for 20–24 h. For knock-down experiments, HeLa cells were seeded and transfected with 2.5 nM siRNA the next day using Lipofectamine2000 (ThermoFisher) following the manufacturer’s protocol. For rescue experiments, after siRNA transfection cells were then split onto 60 mm dishes at 2.5×10^5^ cells/dish, allowed to recover overnight, and then transfected with 1 μg of DNA construct using Lipofectamine and Plus Reagent. HEK-293T cells were seeded in 100 mm dishes at 3×10^6^ cells/dish, cultured for 24 h, and transfected with 4 μg DNA the next day using Lipofectamine and Plus Reagent (ThermoFisher) following the manufacturer’s protocol.

### Lentiviral transduction.

To generate lentivirus, HEK-293T cells were seeded in 100 mm dishes, cultured for 24 h to 80% confluency, and transfected with constructs. 6 μg of construct DNA (pSIN for overexpression or pLKO for shRNA), 4 μg pCMV, and 2 μg pMD2.G were added to 800 μl serum-free DMEM and mixed with 30 μl of PEI. DNA/PEI/DMEM solution was incubated for 15 min at room temperature then added to cells in 12 ml of fresh media and cultured overnight. Media was then changed to 13 ml of complete media, cells were cultured for 24 h, and spent media was collected. To harvest virus, spent media was centrifuged at 1000 rpm for 10 min to remove cell debris, sterile filtered using a 0.45 μm syringe filter. 13 ml media was replaced and after 24 h virus was collected again. Collected virus was combined, mixed, pipetted into 3 ml aliquots, and stored at −80°C. For lentiviral transduction, target cells were seeded in 100 mm dishes at 7×10^5^ cells/dish, cultured overnight, lentivirus with polybrene (1:1000) in complete media was added, and cells were cultured overnight. The next morning, cells were washed with 1x PBS, cultured for 48 h in complete media, then cells were passaged and cells expressing constructs were selected with 2 μg/ml puromycin. After 3–5 days of selection to create semi-stable cells, cells were maintained in complete media supplemented with 1 μg/ml puromycin.

### CRISPR-Cas9 knock-out.

Dock7 was genetically ablated using CRISPR-Cas9 knock-out plasmid and HDR plasmid transfection. Cells were seeded at 2.5×10^5^ cells/well in 6-well plates in antibiotic-free complete media, allowed to recover overnight, then treated. A solution of 1 μg of CRISPR (Santa Cruz Biotechnology, sc-404461) and DOCK7 HDR (h) (Santa Cruz Biotechnology, sc-404461-HDR) plasmid DNA was added to 150 μl Plasmid Transfection Medium (Santa Cruz Biotechnology, sc-108062) was prepared and mixed. A separate solution of 5 μl of UltraCruz Transfection Reagent (Santa Cruz Biotechnology, sc-395739) in 150 μl Plasmid Transfection Medium was also prepared and mixed, then both solutions were incubated for 5 min. Solutions were then mixed, incubated for 15 min, and added to cells. 24 h later, media was changed, cells were cultured for 48 h and then cells expressing plasmid were selected for with 1.75 μg/ml puromycin.

### Immunoblot analysis.

Cells were lysed with cell lysis buffer (50 mM Hepes pH 8.0, 150 mM NaCl, 1 mM MgCl_2_, 25 mM NaF, 1 mM Na_3_VO_4_, 50 mM β-glycerophosphate, 10 μg/ml Leupeptin, 10 μg/ml Aprotinin, and 1% Triton X-100). The lysates were resolved by SDS-PAGE (4–20% Tris-Glycine gels, ThermoFisher), and then the proteins were transferred to polyvinylidene fluoride (PVDF) membranes (PerkinElmer). The membranes were incubated with the indicated primary antibodies diluted in 20 mM Tris pH 7.4, 135 mM NaCl, and 0.02% Tween-20. Primary antibodies were detected with horseradish peroxidase-conjugated secondary antibodies (Cell Signaling Technology) followed by exposure to ECL reagent (PerkinElmer).

### Blue native PAGE.

Blue Native PAGE was performed using lysates prepared from growing HEK-293T cells according to the manufacturer’s protocol (Thermo Fisher). 1×10^7^ cells were collected and lysed using 1x Native PAGE lysis buffer including protease inhibitor cocktail and 1% digitonin. Multiple lanes of the same lysates were then run on one 3–12% Bis-Tris gel followed by denaturing treatment and transferred onto PVDF membrane. Each individual strips of lysates were then blotted for mTOR, Dock7, Raptor, Rictor, TSC1, and TSC2. All antibodies were purchased from Cell Signaling Technology. Blots were developed as described above followed by processing and compilation using ImageJ^85^.

### Soft agar colony formation.

Cells in complete medium (RPMI with 10% FBS) containing 0.3% agarose were seeded onto a layer of 0.6% agarose with complete media in 6-well plates at 8–10×10^3^ cells/well. SK-BR-3, MCF7, and MDA-MB-231 cells were seeded at 5×10^3^, 1×10^4^ and 2×10^4^ cells/well, respectively. Cultures were fed every 3–4 days with complete medium containing 0.3% for 14–21 days. At endpoints, 1 mg/ml NBT in 1x PBS was added to agar, cultured overnight, and then imaged.

### Cell growth assay.

Cells were seeded in 6-well plates at 2×10^4^ cells/well and cultured overnight in complete media. The next day, media was changed to serum-free DMEM with fresh media replaced every other day and after either two days or four days cells were counted.

### EdU cell proliferation assay.

DNA synthesis was directly measured in live cells with the EdU Staining Proliferation kit iFluor 488 (Abcam, ab219801) following the manufacture protocol. Cells were seeded in 6-well plates with no. 1.5 22×22 mm coverslips (Electron Microscopy Sciences, 72204–01) at 1.5×10^5^ cells/well, cultured for 48 h, then washed with 1x PBS, and treated with complete or serum-free media. After 20–24 h incubation, cells were incubated with 10 μm EdU solution for 4 h, fixed in 4% paraformaldehyde (PFA; ThermoFisher, J19943.K2) for 10 min, permeabilized, and labeled using kit components. Coverslips were mounted using Vectashield with DAPI (Vector, H-1200) microscope slides and sealed with nail polish. Analysis was performed in ImageJ using the Particle Analyzer plugin and proliferation was calculated as the fraction of EdU positive cells.

### Apoptosis Assay.

The In Situ Cell Death Detection Kit, Fluorescence (Roche, 11684795910) was used to label quantify DNA strand breaks with TUNEL according to the manufactures protocol. Cells were seeded in 6-well plates with no. 1.5 22×22 mm coverslips (Electron Microscopy Sciences, 72204–01) at 1.5×10^5^ cells/well, grown for 48 h, the washed with 1x PBS, and treated with complete or serum-free media for 18–24 h. After incubation, media was removed and cells were washed 3x with 1x PBS, fixed in 4% PFA (ThermoFisher, J19943.K2) for 10 min, washed 3x with 1x PBS, and stored in 1x PBS at 4°C until use. After TUNEL, immunostaining was performed. Samples were washed 3x with 0.02% Tween20 (Sigma-Aldrich, P1379) in 1x PBS for 15 min each, blocked using 3% BSA/10% FBS/0.02% Tween20 in 1x PBS for 3 h, and incubated with primary antibody for Cleaved Caspase-3 (1:200, Cell Signaling Technology) overnight at 4°C. Samples were then washed 3x for 15 min each with 0.02% Tween20, goat anti-rabbit AlexFluor594 (1:200, Invitrogen) in blocking solution was added for 2 h at room temperature, and washed 3x for 15 min each with 0.02% Tween20. Coverslips were mounted using Vectashield with DAPI (Vector, H-1200) microscope slides and sealed with nail polish. Analysis was performed in ImageJ using the Particle Analyzer plugin. Apoptotic cells were identified as cells positive for both TUNEL and Cleaved Caspase 3.

### Isolation of Mouse Embryo Fibroblasts (MEF).

Pregnant mouse was euthanized as approved by AICUC 14 days after appearance of copulation plug. Uterus was removed and embryos were extracted and placed in 60mm tissue culture dishes, head was removed and used for genotyping, and internal organs were discarded. Each embryo was washed with HBSS, with no calcium nor magnesium (ThermoFisher #14170120) twice, and then placed in 0.25% trypsin-EDTA (Corning, #25053CI). Embryonic tissue was minced into small pieces, and placed in 5% CO2, 37°C incubator for 5mins. Mixture was pipetted up and down and placed back in incubator for 10mins. Trypsin was deactivated with 15mL DMEM, 10% FBS, and transferred to a 50mL tube where it rested for 10mins. Transfer the single cell and cell cluster supernatant to a 100mm tissue culture plate and let cells attached overnight. Change media to DMEM, 10% FBS the next day, and cells will be ready for use two days after.

### Immunoprecipitation.

HEK-293T cells were lysed with lysis buffer (50 mM Hepes pH 8.0, 150 mM NaCl, 1 mM MgCl_2_, 25 mM NaF, 1 mM Na_3_VO_4_, 50 mM β-glycerophosphate, 10 μg/ml Leupeptin, 10 μg/ml Aprotinin, 0.3% CHAPS). Lysates were pre-cleared with BSA-coated Protein G beads (ThermoFisher) on a rotator at 4°C for 15 min. The supernatant was collected and added with anti-Myc (Covance), HA (Covance), or Flag (Sigma-Aldrich) antibody for 2 h, then BSA-coated Protein G beads were added and incubated for 1 h at 4°C. Immunoprecipitates were washed 3x with lysis buffer followed by the addition of 2x Laemilli buffer. For nucleotide binding to HA-tagged Cdc42, Rac1, and Rheb, cells were transfected and lysed as described above. Cell lysates were then treated with 10 mM EDTA, 10 mM EDTA + 1 mM GDP, or 10 mM EDTA + 100 μM GTPγS at room temperature for 15 min. 50 mM MgCl_2_ was then added to samples containing nucleotides followed by immunoprecipitation as described above.

### Nucleotide-dependent GST fusion protein pull-down.

GST and GST-Rheb were expressed in BL21 cells and purified by affinity chromatography using Glutathione Sepharose High Performance beads (GE Healthcare Life Sciences) according to the manufacturer’s protocol. GST and GST-Rheb were stored on the glutathione beads with 30% glycerol at −20°C until use. HEK-293T cells were infected with lentivirus containing constructs overexpressing either DHR1-V5 or DHR2-V5. 48 h after infection, cells were selected with 2 μg/mL puromycin for 48 h to create semi-stable cell lines of DHR1-V5 and DHR2-V5. Cells were maintained in 1 μg/mL puromycin after selection. Whole cell lysates of DHR1-V5 and DHR2-V5 were lysed with cell lysis buffer (50 mM Hepes pH 8.0, 150 mM NaCl, 1 mM MgCl_2_, 25 mM NaF, 1 mM Na_3_VO_4_, 50 mM β-glycerophosphate, 1 mM DTT, 10 μg/ml Leupeptin, 10 μg/ml Aprotinin, 1% Triton X-100). Lysates were precleared using GST beads. GST-Rheb was treated with 10 mM EDTA, 10 mM EDTA + 1 mM GDP, or 10 mM EDTA + 100 μM GTPγS at RT for 15 min followed by the addition of 50 mM MgCl_2_ for the samples containing nucleotides. GST controls were either treated with EDTA or non-nucleotide loaded (no EDTA). Nucleotide-loaded or nucleotide-free GST/GST-Rheb were added to the precleared lysates of DHR1-V5 or DHR2-V5. Pull-downs were done in the lysis buffer ± EDTA at 4 °C for 2 h. Pulled-down proteins were washed 3x with the lysis buffer ± EDTA, 2x Laemilli buffer was added, and analyzed on 12% Tris-Glycine gels.

### Western blot quantification.

Western blots were quantified using ImageJ under the Gel Analysis Tool^86^. The intensity of the different lanes was then normalized to the control lane, which was set to one. For phospho-protein signals, the intensities of each phospho-protein were first divided by the intensities of the total protein then the control lane was set to one. The percentage of recovery is calculated by (Intensity_rescue_ − Intensity_knock-down_)/ (1 − Intensity_knock-down_) × 100%.

### Proximity ligation assay (PLA).

PLA was used to examine endogenous protein-protein interactions. Cells were seeded in 6-well plates with no. 1.5 22×22 mm coverslips (Electron Microscopy Sciences, 72204–01) at 1.5×10^5^ cells/well. Cells were grown in complete media for 48 h, washed with 1x PBS, and treated with complete or serum-free media for 20–24 h. After incubation, media was removed and cells were washed 3x with 1x PBS, fixed in 4% paraformaldehyde (ThermoFisher, J19943.K2) for 10 min, washed 3x with 1x PBS, and stored in 1x PBS at 4°C until use. Samples were permeabilized with 0.1% Triton X-100 (Millipore, 1.08603.1000) for 15 min, washed 3x with 0.02% Tween20 (Sigma-Aldrich, P1379) in 1x PBS for 15 min each, and blocked using PLA Blocking Solution for 30 min at 37°C prior to PLA. Duolink In Situ PLA Probe Anti-Mouse PLUS (Sigma-Aldrich, DUO92001), Duolink In Situ PLA Probe Anti-Rabbit Minus (Sigma-Aldrich, DUO92005), In Situ Detection Reagents Red (Sigma-Aldrich, DUO92008) were used to examine endogenous protein-protein interactions following the Duolink PLA Fluorescence protocol. Target proteins were detected using primary antibodies for Dock7 (1:200), AKT (1:200), AKT (1:200), p-AKT S473 (1:50), TSC2 (1:200) mTOR (1:200), Cdc42 (1:200), PHLPP (1:50), and PP2A/B (1:50). Dock7 antibody was purchased from Santa Cruz Biotechnology, PHLPP was purchased from Proteintech, and all other antibodies were obtained from Cell Signaling Technologies. PLA multicolor was used following the Duolink Multicolor Detection protocol. Primary antibodies for V5-tag (Invitrogen, MA5–15243) and pan AKT (R&D Systems, MAB2055) conjugated to Duolink PLA Multicolor Probemaker Kit Green (Sigma Aldrich, DUO96020), while pan AKT (R&D Systems, MAB2055) and p-AKT S473 (R&D Systems, AF887) were conjugated with the Probemaker Kit Red (Sigma-Aldrich, DUO96010). Duolink PLA Multicolor Reagent Pack (Sigma-Aldrich, DUO96000) was then used for amplification and detection. In Situ Wash Buffers Fluorescence (Sigma-Aldrich, DUO82049) were used for all wash steps in PLA procedures. Coverslips were mounted on a microscope slide with Vectashield mounting media with DAPI (Vector, H-1200) and sealed for imaging.

### Fluorescence microscopy.

Fluorescence images was performed on a Keyence BZ-X810 inverted fluorescence phase contrast microscope using a Plan Apochromat 60x/1.4 NA oil immersion objective (BZ-PA60, Keyence Corp) and ET DAPI (ex. 395/25 em. 460/50; Chroma, 4900-UFI) ET EGFP (ex. 470/40 em. 525/50; Chroma, 49002-UFI), and ET mCH/TR (ex. 560/40 em. 630/75; Chroma, 49008-UFI) filters was used for fluorescence imaging. PLA Images were taken with 2x digital zoom, optical sectioning (1D, width=10), and z-stacks (0.5 μm pitch) taken through the height of the cells.

### TCGA Data.

The Cancer Genome Atlas (TCGA) Breast Cancer (BRCA) data set was assessed and analyzed through UCSC Xena browser^87^.

### Statistical analysis.

All statistical analysis was performed using GraphPad Prism 9.0. Normality in the data spread was tested with the D’Agostino-Pearson omnibus normality test. Data with a Gaussian distribution were compared using a two-tailed Student’s t-test for two groups and one-way ANOVA with Dunn’s post hoc analysis for multiple groups. Non-parametric tests were used to compare data with a non-normal distribution and a two-tailed Mann-Whitney test was used for two groups, while one-way Kruskal-Wallis test was used for multiple groups. No statistical method was used to determine sample size. All experiments were reproduced at least three independent times.

## Supplementary Material

Supplement 1
