# A multiprotein signaling complex sustains AKT and mTOR/S6K activity necessary for the survival of cancer cells undergoing stress

**DOI:** 10.1101/2023.01.03.522657

**Published:** 2024-02-27

**Authors:** Oriana Y. Teran Pumar, Matthew R. Zanotelli, Miao-chong Joy Lin, Rebecca R. Schmitt, Kai Su Green, Katherine S. Rojas, Irene Y. Hwang, Richard A. Cerione, Kristin F. Wilson

**Affiliations:** 1 Department of Molecular Medicine, Cornell University, Ithaca, NY 14853, USA; 2 Department of Chemistry, Cornell University, Ithaca, NY 14853, USA

**Keywords:** cancer, cell survival, Dock7, Cdc42, mTOR-AKT

## Abstract

Cancer cells encounter stresses during tumor progression and metastatic spread, however, how they survive these challenges is not fully understood. We now identify a mechanism for cancer cell survival through the discovery of a multiprotein signaling complex that includes the GTPase Cdc42, the Cdc42 GEF/effector protein Dock7, AKT, mTOR and the mTORC1 regulatory partners TSC1, TSC2, and Rheb. This pro-survival signaling complex sustains the activated state of AKT by preventing its dephosphorylation at Ser473 during serum starvation, resulting in a low but critical activation of a Raptor-independent mTOR/S6K activity. We demonstrate that the Dock7 DHR1 domain, previously of unknown function, is responsible for preserving AKT phosphorylation through an interaction requiring its C2-like motif. Collectively, these findings help address long-standing questions of how Cdc42 signals mTOR activation by elucidating the unique functions of its signaling partner Dock7 as an AKT regulator necessary for resistance to anoikis and apoptosis in cancer cells.

## Introduction

As critical regulators of cell proliferation, growth, and survival, members of the mechanistic target of Rapamycin (mTOR) pathway are often exploited in cancer.^1-5^ mTOR signaling is hyperactivated in most cancers through numerous mechanisms, such as the amplification of receptor tyrosine kinases that signal to phosphatidylinositol-3-kinase (PI3K) (e.g., HER2 and EGFR), hyperactivation of AKT (Protein Kinase B), gain of function mutations in the catalytic subunit of PI3K (*PIK3CA*), or deletions in PTEN, one of the main negative regulators of the PI3K/mTOR pathway.^6-8^ Upon oncogenic transformation, cancer cells proliferate rapidly, doubling their lipid, nucleotide, and protein content with each cell division. Anabolic processes are thus enhanced and catabolic processes are altered to meet the biosynthetic demands of rapidly growing cancer cells, and these requirements are often met by the activation of signaling pathways that activate mTOR.^9-13^ However, the function and regulation of these pathways in stress conditions such as the absence of growth factors and other nutrients remains largely unknown.

mTOR is a serine/threonine kinase and a member of the PIKK family which is known to function within two distinct complexes, mTORC1 and mTORC2.^14-16^ mTORC1 is a Rapamycin-sensitive, nutrient and mitogen-sensing complex defined by its interaction with the accessory protein Raptor.^17, 18^ mTORC1 has been classically studied in the context of growth factor and amino acid signaling and shown to be recruited to the lysosome by the interaction between Raptor and the heterodimeric GTPase RagA/B-C/D complex, which is activated by the Ragulator-v-ATPase.^19-21^ When bound to the lysosome, mTORC1 then interacts with its direct activator the small GTPase Ras homolog enriched in brain (Rheb).^22^ Rheb directly stimulates mTORC1 kinase activity which triggers anabolic processes, including protein and lipid synthesis, while inhibiting catabolic processes such as autophagy. AKT regulates mTOR activity, with mTORC1 being a downstream effector of AKT and mTORC2 being an upstream regulator. AKT catalyzes the phosphorylation of tuberous sclerosis complex (TSC) 2 at Thr1462, that together with TSC1 forms a GTPase Activating Protein (GAP) complex for Rheb.^23^ The AKT-catalyzed phosphorylation of TSC2 disables the GAP activity of TSC1/TSC2, enabling Rheb to activate mTORC1.^24-27^ Unlike mTORC1, mTORC2 is not inhibited by acute Rapamycin treatment nor does it require Rheb to stimulate its kinase activity.^28-30^ mTORC2 consists of mTOR, Rictor, mLST8, and mSin1, and has been shown be activated by multiple mitogenic stimuli as well as promote cell survival in response to stresses such as the deprivation of growth factors and nutrients.^31, 32^ AKT, a major substrate of mTORC2, is phosphorylated within its hydrophobic motif at Ser473, activating its ability to phosphorylate and inhibit numerous pro-apoptotic proteins, such as Bad and Caspase-9, thereby blocking apoptosis.^33^ AKT is widely regarded as a quintessential regulator of cell survival^34-36^ and is one of the most activated protein kinases in human cancer, with AKT hyperactivation associated with resistance to apoptosis.^37-39^ Still, the mechanisms that enable AKT in nutrient-deprived cells to remain active and avoid dephosphorylation by phosphatases have remained elusive.

While Rheb and the Rags have well established roles in mTORC1 activity, members of the Rho GTPase family have also been implicated in regulating mTOR signaling during important biological processes such as cap-dependent mRNA splicing and neurogenesis.^40-45^ Like all small GTPases, Rho GTPase activation-deactivation cycles are regulated by GAPs which enhance their intrinsic GTPase activity to deactivate their signaling capability, and by Guanine nucleotide Exchange Factors (GEFs) that catalyze the dissociation of GDP and allow for the more abundant GTP in cells to bind and induce their signaling-active states.^46-48^ Rho GTPases are a subclass of the Ras superfamily including RhoA, Rac1 and Cdc42, and are traditionally known for their roles in cytoskeletal remodeling.^49^ However, there is increasing evidence implicating the improper regulation of Rho GTPases in driving tumorigenesis.^50-52^ A mutant of Cdc42 that is capable of rapidly exchanging GTP for GDP and other constitutively active forms of Rho GTPases have been shown to induce transformation by supporting cellular growth under serum deprivation, anchorage-independent colony formation, and *in vivo* tumor formation.^53, 54^ Despite the signaling activities of both Rho GTPases and mTOR playing important roles in tumorigenesis and the various indications that Rho GTPases and their GEFs can stimulate mTOR activation, the mechanisms linking these two processes have been poorly understood.

Here, we identify a unique multiprotein complex that includes Cdc42 and the Cdc42/Rac GEF and Cdc42-signaling effector, Dock7, that can protect and maintain low levels of AKT activation as well as promote a growth factor-independent activation of mTOR/S6K. Dock7 serves as a binding partner for AKT, mTOR, and other mTOR-associated proteins including TSC1/2 and Rheb, thus enabling these signaling proteins to form a complex and work together to provide a pro-survival signaling activity. We find that Dock7 sustains a low level of AKT phosphorylation and stimulates a Raptor-independent mTOR/S6K activity during serum deprivation, and is required for cancer cell survival, anchorage-independent growth, and an enhanced transformed phenotype. Dock7 belongs to the atypical Dock180 family of Rho GEFs with two conserved Dock Homology Regions (DHR) 1 and DHR2.^55, 56^ DHR2 contains a well-studied GEF domain that activates either Cdc42 or Rac1, and a putative dimerization region, while DHR1 has a C2-like motif ascribed to membrane phospholipid binding in other Dock proteins, but has no reported signaling function.^57, 58^ Surprisingly, we find that the GEF activity of Dock7 is dispensable for its ability to work together with Cdc42 to sustain AKT activation and stimulate mTOR/S6K activity, while the DHR1 domain through its C2-like motif is essential for these signaling outcomes.

## Results

### Cdc42 works together with its signaling partner Dock7 to activate mTOR.

**Figure 1.**
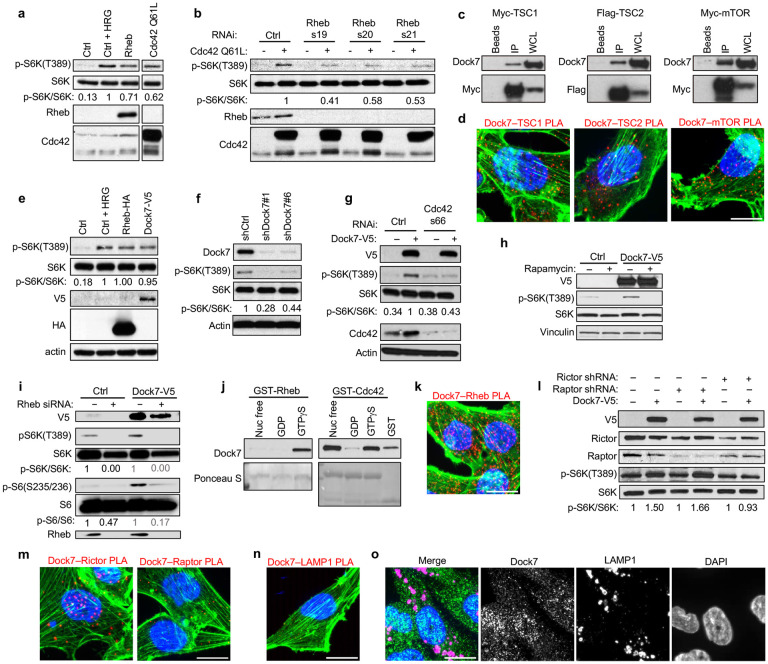
Dock7 interacts with TSC1, TSC2, Rheb, and mTOR and stimulates S6K in a Cdc42-dependent manner that requires Rictor, but not Raptor. (**a**) Western blot of HeLa cells that were transiently transfected with HA-tagged WT Rheb or Cdc42 Q61L and serum starved overnight. Heregulin (HRG) treatment (1 nmol/L for 1 h) was used as a control to confirm mTORC1 signaling. (**b**) Western blot reading out the ability of transiently overexpressed Cdc42 Q61L to activate S6K in serum starved HeLa cells in the presence or absence of Rheb. (**c**) Western blot of endogenous Dock7 co-immunoprecipitating with ectopically expressed Flag-TSC2, Myc-TSC1, or Myc-mTOR from HEK-293T cells grown in complete media. (**d**) PLA images of interactions between endogenous Dock7 and TSC1, TSC2, or mTOR in MDA-MB-231 grown in serum free media. (**e**) HeLa cells transiently transfected with either Dock7 or Rheb and starved overnight. HRG treated (1 nmol/L, 30 min) and mock-transfected cells were used as positive and negative controls, respectively. (**f**) Western blot showing S6K activity in serum starved MDA-MB-231 cells following Dock7 knockdown. (**g**) Western blot depicting S6K activity in serum starved HeLa cells when V5-tagged Dock7 was transiently overexpressed, and Cdc42 was knocked down using siRNA. (**h**) Western Blot showing S6K activity in serum starved HeLa cells transiently expressing Dock7-V5 in presence of either a vehicle control (DMSO, 1:1000) or Rapamycin (1 nM). (**i**) Western blot of the phosphorylation of either S6K or S6 in serum starved HeLa cells transiently overexpressing Dock7-V5 in the presence of absence Rheb knockdown using siRNA.(**j**) Recombinant GST-tagged Rheb proteins loaded with either GTPγS, GDP, or EDTA-treated were used to pull down endogenous Dock7 from HEK-293T cells. Blots were probed with anti-Dock7 antibody to determine small GTPase-Dock7 binding. Ponceau S was used to visualize the GST proteins. Pulldowns with Cdc42-GST proteins were used as control. (**k**) PLA image of interactions between endogenous Dock7 and Rheb in MDA-MB-231 cells grown in serum free media. (**l**) Either Rictor or Raptor were semi-stably knocked down from HeLa cells using shRNA, and then full-length V5-tagged Dock7 protein was overexpressed. After 48 h, cells were starved overnight, and lysates were collected for Western blotting to determine S6K activation. (**m**) PLA images of interactions between Dock7 and Rictor and Dock7 and Raptor in MDA-MB-231 cells grown in serum free media. (**n**) PLA image of interactions between endogenous Dock7 and LAMP1 in MDA-MB-231 cells grown in serum free media. (**o**) Immunofluorescence images of Dock7 and LAMP1 in MDA-MB-231 cells grown in serum free media.

It has been known for some time that Rho family GTPases are able to stimulate mTOR activity with important consequences in various biological contexts.^40-45^ For example, Cdc42 has been reported to bind and mediate the growth factor-induced activation of S6K,^40^ a frequently used read-out for mTOR stimulation.^59^ However, the mechanisms of activation have not been clearly elucidated. Indeed, we find ectopic expression of the constitutively active form of Cdc42 (Cdc42 Q61L) in HeLa cells stimulated S6K phosphorylation at Thre389 (p-S6K), comparable to the activation by Heregulin (an activator of ErbB2/HER2) or upon ectopic expression of the small GTPase Rheb, which directly activates mTORC1^22^ (Fig. 1a). Knockdown of Rheb attenuated, but did not completely eliminate the ability of constitutively active Cdc42 to signal to S6K in serum deprived cells, (Fig. 1b), suggesting that Cdc42 can signal to S6K through more than one mechanism.^40^ Here, we identify a growth factor-independent Cdc42-mTOR pathway that plays a critical role in the ability of cancer cells to resist severe stresses such as those imposed by nutrient deprivation.

A possible clue regarding how Cdc42 may signal to mTOR came from an earlier report suggesting that Dock7, a member of the Dock180 family of Rho-GEFs that we have shown to be both a GEF and an effector for Cdc42,^60^ is capable of interacting with the Rheb GAP complex TSC1-TSC2 that functions as a negative regulator of mTORC1 activity.^61^ To establish that Dock7 can in fact interact with TSC1/2, we transiently transfected HEK-293T cells with either epitope-tagged TSC1 or TSC2, and isolated these proteins via immunoprecipitation. Endogenous Dock7 co-immunoprecipitated with both Myc-tagged TSC1 and TSC2, and we also found that Myc-mTOR was capable of co-immunoprecipitating endogenous Dock7 (Fig. 1c), suggesting that Dock7 forms complexes with TSC1, TSC2, and mTOR. Consistent with these observations, Dock7 co-migrated with mTOR, TSC1, and TSC2 as a high molecular weight species upon performing Blue Native-PAGE (BN-PAGE) using HEK-293T cell lysates,^62^ a technique used to characterize the components of large complexes based on their native molecular mass (Supplemental Fig. S1). We then examined protein interactions with a complementary approach using proximity ligation assays (PLA), which can quantitatively visualize protein-protein interactions within tens of nanometers as puncta *in situ*.^63, 64^ PLAs in the breast cancer cell line MDA-MB-231 yielded robust puncta and verified interactions between endogenous Dock7 and TSC1, TSC2, and mTOR, demonstrating that these complexes can form in multiple cell lines (Fig. 1d). Like activated Cdc42, the ectopic expression of Dock7 promoted S6K phosphorylation during serum starvation, and the knockdown of Dock7 reduced basal levels of S6K phosphorylation (Fig. 1e,f). Additionally, Dock7 required Cdc42 to activate S6K, identifying a previously unidentified role for Dock7 in a Cdc42-dependent regulation of mTOR activation (Fig. 1g).

### Dock7 stimulation of mTOR/S6K activity requires both mTORC2 and Rheb.

Given that Dock7 associated with mTOR and TSC1/2, as well as stimulated the phosphorylation of S6K, we sought to further define the mechanism by which Dock7 mediates the activation of an mTORC1-like activity in nutrient-deprived cells. Treatment with Rapamycin, an mTORC1-specific inhibitor, blocked the ability of ectopically expressed Dock7-V5 to promote the phosphorylation of S6K (Fig. 1h), and knocking down Rheb prevented Dock7 from stimulating the phosphorylation of both S6K and ribosomal S6, the major target of S6K (Fig.1i). Rheb, specifically when in an activated state bound to the GTP analog GTPγS, was capable of associating with Dock7 in pull-down experiments, whereas both nucleotide-free and GTPγS-bound forms of Cdc42 associated with Dock7, consistent with its role as both a Cdc42-GEF and a Cdc42 signaling effector^60^ (Fig. 1j). GST-pull down assays were supported by PLA, which showed a substantial number of interactions between endogenous Dock7 and Rheb during serum starvation (Fig. 1k). These data indicate an involvement of Rheb in this complex to facilitate a Dock7-dependent mTORC1-like activity.

mTOR exists in two distinct complexes, mTORC1 and mTORC2,^14-16^ each with different functions that are known to undergo crosstalk through feedback mechanisms.^65, 66^ Thus, we attempted to delineate the role of mTORC1 and mTORC2 in the ability of endogenous or ectopically expressed Dock7 to activate S6K in serum-starved cells in which either the mTORC1-defining subunit Raptor, or the mTORC2-specific subunit Rictor, was knocked down. The knockdown of Raptor unexpectedly showed no effect on the phosphorylation of S6K under the conditions tested, whereas knocking down Rictor resulted in significant reductions in S6K phosphorylation (Fig. 1l). S6K is not a direct substrate of mTORC2; therefore, the Rictor-dependent phosphorylation of S6K suggests Dock7 acts downstream of mTORC2. We examined Dock7 associations with Raptor and Rictor by co-immunoprecipitation and PLA. While both ectopically expressed Rictor and Raptor associated with mTOR as predicted, neither Rictor nor Raptor demonstrated a robust ability to co-immunoprecipitate with Dock7 when isolated from growing HEK-293T cells (Supplemental Fig. S2). However, we did observe many robust PLA interactions between endogenous Dock7 and Rictor, but only a small number of weak interactions between Dock7 and Raptor in serum starved MDA-MB-231 cells (Fig. 1m), suggesting that Rictor specifically may be capable of associating with the Dock7 complex. Considering that Raptor facilitates the lysosomal localization of mTOR, which is essential for the canonical nutrient-sensing activation of mTORC1,^19^ the absence of Raptor prompted us to investigate whether Dock7 could undergo a Raptor-independent localization to the lysosome. PLA and immunofluorescence showed no association or co-localization of Dock7 with the endolysosome marker LAMP1 during serum deprivation, suggesting that Dock7 stimulates S6K activity independently of lysosomal involvement (Fig. 1n,o). Together, these observations suggests that Dock7 functions downstream of mTORC2 to regulate a mTORC1-like activity which is distinct from canonical mTORC1.

### Dock7, together with mTOR, AKT and S6K, promotes signaling activities essential for cancer cell survival.

**Figure 2.**
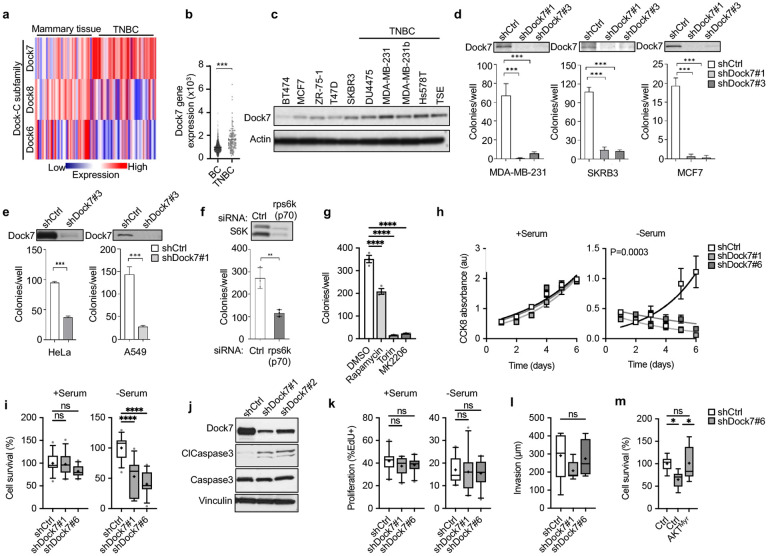
Dock7 is required for the transformation and survival of cancer cells and suggests functional roles for mTOR, AKT and S6K. (**a**) The Cancer Genome Atlas (TGCA) expression profile for Dock-C family members in triple-negative breast cancer (TNBC) and mammary tissue. (**b**) Dock7 mRNA levels across patients with either receptor-positive (BC) or triple-negative breast cancers (TNBC). (**c**) Western blot indicating protein expression levels of Dock7 across breast cancer cell lines. (**d**) Soft agar colony formation of MDA-MB-231, SKRB3 and MCF7 breast cancer cells with or without the knockdown of Dock7. (**c**) Soft agar colony formation of HeLa and A549 cells with or without the knock-down of Dock7. (**f**) Soft agar colony formation of HeLa cells following S6K knockdown using siRNA. (**g**) Soft agar colony formation of HeLa cells grown in complete media containing either vehicle control (DMSO, 1:1000), Rapamycin (1 nM), Torin (100 nM), or MK2206 (10 μM). Colonies were quantified 2 weeks after first drug treatment. (**h**) Cell viability was measured by CCK8 absorbance over 6 days in Dock7 knockdown MDA-MB-231 cells in the presence of absence of serum. (**i**) Cell survival assay of MDA-MB-231 cells following Dock7 knockdown and assessing cell count after 2 days in full serum or serum-free media. (**j**) Western blot analysis of Cleaved Caspase 3 from HeLa cells where Dock7 was knocked down using shRNA. (**k**) Cell proliferation assays performed in the presence or absence of serum for MDA-MB-231 cells following Dock7 knockdown and as determined by EdU incorporation. (**l**) MDA-MB-231 breast cancer cell spheroid invasion in collagen over 24-48 h post embedding. (**m**) Cell survival assay in serum starved Dock7 knockdown MDA-MB-231 cells with or without the overexpression of a constitutively active AKT mutant construct (AKT^Myr^). Data shown as median ± interquartile range (box), 5–95 (whiskers), and mean (+), or mean ± s.d. *P<0.05, **P<0.001, ***P<0.0001, ns = not significant.

Both mTOR and Rho GTPase GEF signaling are hyperactivated in cancer.^1-5, 50-52^ Searching the Cancer Genome Atlas (TCGA) Breast Cancer Dataset (BRCA) we found that unlike other related members of the Dock180 family, Dock7 is highly upregulated in triple-negative breast cancer patients (Fig. 2a,b). Examining Dock7 protein expression levels among a panel of 10 different breast cancer cell lines, half of which were HER2^+^, ER^+^/PR^+^/HER2^+^, or ER^+^/PR^+^ cell lines, while the remaining half were triple-negative breast cancer cell lines, also showed that triple-negative breast cancer cells had higher Dock7 protein expression. (Fig. 2c). Given the relevance of Dock7 in cancer, we first investigated its impact on oncogenic transformation by examining the effects on anchorage-independent growth, a well-known phenotype of malignant cells.^67^ Upon knocking down Dock7, we observed a striking decrease in the ability of triple-negative MDA-MB-231 cells, as well as receptor-positive SKBR3 and MCF7 breast cancer cells, to form colonies in soft agar (Fig. 2d). The knockdown of Dock7 also impacted the ability of HeLa cervical cancer cells and A549 lung cancer cells to form colonies (Fig. 2e), establishing a role for Dock7 in the transformed properties of multiple cancer cell types.

Given the physical and signaling relationships demonstrated above between Dock7, mTOR and S6K as well as the role of mTOR/S6K in tumorigenesis,^68^ we examined how these activities might relate to the role of Dock7 in transformation. We found that both the knockdown of S6K and the treatment of HeLa cells with Rapamycin resulted in an approximately 50% decrease in the formation of colonies in soft agar (Fig. 2f,g). As Rictor-associated mTOR activity proved important in Dock7 signaling, we also probed the contributions of mTORC2 and its signaling target, AKT, to the anchorage independent growth of HeLa cells by treating them with Torin, a pan mTOR inhibitor, or MK2206, an AKT inhibitor, and found that both treatments fully blocked growth in soft agar (Fig. 2g). Collectively, these results suggest a signaling cascade whereby mTORC2 activates AKT to promote transformation and that mTORC1-like activity/S6K would represent a significant outcome of the mTORC2/AKT signaling.

AKT promotes cell survival, proliferation, and invasion^69^ and is a negative regulator of apoptosis,^70, 71^ so we examined the role of Dock7 in each of these transformed phenotypes. The knock-down of Dock7 specifically lowered cell viability as measured by CCK8 during serum-free culture conditions compared to complete medium (Fig. 2h). Similarly, the ability of MDA-MB-231 cells to survive in serum-free media was significantly impacted by the loss of Dock7 while cells grown in complete media were not affected (Fig. 2i). Apoptosis, as assessed by increases in the levels of the apoptotic marker, cleaved caspase-3, was elevated in serum-starved HeLa cells where Dock7 was knocked down (Fig. 2j). In contrast, the knock-down of Dock7 did not influence cell proliferation rates regardless of culture conditions (Fig. 2k). The expression of Dock7 was also dispensable for the invasive activity of MDA-MB-231 cells, as measured by spheroid invasion in collagen (Fig. 2l). Finally, the Dock7-dependent loss of cell survival during serum deprivation could be rescued by ectopically expressing a myristoylated constitutively active form of AKT (AKT^Myr^) (Fig. 2m), suggesting that Dock7 may play a role in regulating an AKT activity during nutrient deprivation which is critical to conferring a survival advantage to cancer cells. Together, these findings show that while Dock7 is not required for mitogenic growth, it maintains a necessary level of AKT activity that imparts an essential survival function when cancer cells are challenged with environmental stress.

### Dock7 protects AKT from dephosphorylation at Ser473.

**Figure 3.**
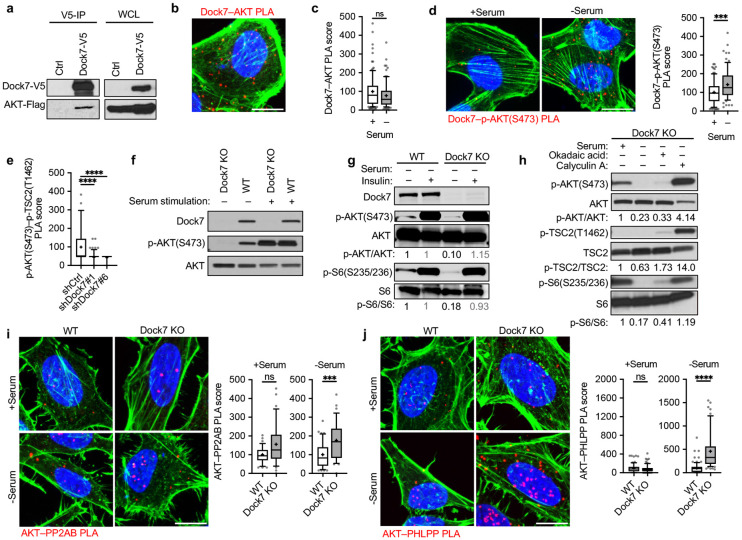
Dock7 protects AKT from dephosphorylation to enhance cancer cell survival during serum deprivation. (**a**) Immunoprecipitation of transiently overexpressed Flag-tagged AKT and Dock7-V5 in HEK-293T cells resolved on an SDS-PAGE gel for Western analysis. (**b**) PLA image of interactions between endogenous Dock7-AKT in MDA-MB-231 cells. (**c**) Dock7–AKT PLA in MDA-MB-231 cells in complete media or following overnight serum starvation. (**d**) Dock7–p-AKT(S473) PLA in MDA-MB-231 cells in complete media or following overnight serum starvation. (**e**) p-AKT(S473)–p-TSC2(T1462) PLA interactions in MDA-MB-231 cells in complete media or following overnight serum starvation. (**f**) HeLa cells were seeded and allowed to recover for a day before changing media to serum-free media for 24 h, serum restimulation (10% FBS) was performed with dialyzed serum for 1 h prior to collection and lysis. Western blotting indicates effects of p-AKT(S473) (**g**) HeLa cells were seeded and allowed to recover for a day before changing media to serum-free media for 24 h. Cells were treated with and without 100 nM insulin for 1 h before being collected and used for Western blot analysis determine effects on the status of p-AKT(S473) or p-S6(S235/236). (**h**) Crispr-Cas9 Dock7 KO HeLa cells were seeded and allowed to recover for 24 h. Media was then changed to either full serum media or serum-free media for 24 h. Serum-starved cells were then treated with either vehicle control (DMSO 1:1000), Okadaic Acid (10 nM), or Calyculin A (50 nM) for 1 h before being collected for Western blot analysis to indicate the phosphorylation status of AKT, TSC2 and S6. (**i, j**) PLA was performed on Cirspr-Cas9 Dock7 KO and WT HeLa cells to measure the number of complexes formed by AKT with (**h**) PHLPP and (**i**) PP2AB in the presence and absence of serum. Data shown as median ± interquartile range (box), 5–95 (whiskers), and mean (+), or mean ± s.d. *P<0.05, **P<0.001, ***P<0.0001, ns = not significant.

Given the possibility that Dock7 helps to maintain the activation of AKT in the absence of serum, we examined the ability of Dock7 to interact with AKT. Co-immunoprecipitation experiments utilizing the co-expression of V5-tagged Dock7 and Flag-tagged AKT in growing HEK-293T cells showed that a relatively small but detectable amount of transiently expressed AKT co-precipitated with Dock7-V5 under these conditions (Fig. 3a). We then used PLA to confirm that Dock7 was in sufficient proximity to interact with AKT *in situ* (Fig. 3b). Interestingly, it appears by PLA that Dock7 was in proximity to interact with AKT regardless of serum conditions (Fig. 3c), whereas the probability of interactions between Dock7 and AKT phosphorylated at Ser473 were enhanced upon the removal of serum (Fig. 3d). Dock7 appeared to be necessary for maintaining interactions between active AKT and its target, TSC2, as knocking down Dock7 significantly reduced interactions between endogenous p-AKT(S473) and p-TSC2(T1462) (Fig. 3e).

To further study how AKT activity might be maintained by Dock7 during serum deprivation, we created a Dock7 knock-out (KO) model using Crispr-Cas9 to better control for the residual effects of partial knockdowns. The knockout of Dock7 was lethal in MDA-MB-231 cells, but we were able to generate a Dock7 KO HeLa cell line which showed a dramatic reduction in soft agar colony formation, like knock-down experiments (Supplemental Fig. S3a). Like the case when knocking down Dock7, we saw that Dock7 KO cells were unable to survive serum free conditions relative to wild-type (WT) cells and displayed enhanced rates of apoptosis (Supplemental Fig. S3b,c). Proliferation was unaffected by the Dock7 KO (Supplemental Fig. 3d). Interestingly, Murine Embryonic Fibroblasts (MEFs) derived from Dock7 knock out mice did not experience a decrease in survival when grown in serum-free conditions, which suggests Dock7 may only provide a survival benefit in cancer cells (Supplemental Fig. S3e).

We next used the Dock7 KO cells to further examine Dock7-dependent AKT signaling and identify how Dock7 sustained AKT activity during serum starvation. In both Dock7 KO and WT cells, serum and insulin stimulated AKT phosphorylation at Ser473 and the S6K substrate S6 at Ser235/236 to the same robust extents; however, the basal phosphorylation levels of both AKT and S6 were diminished specifically in serum starved KO cells (Fig. 3f,g). This suggested that Dock7 might protect AKT from phosphatase activity when cancer cells are nutrient deprived. As a first approach to examine this question, Dock7 KO cells were cultured in either full-serum or serum-free media overnight and treated with Okadaic Acid or Calyculin A, inhibitors of serine/threonine protein phosphatases.^72^ Upon inhibition of phosphatase activity with Calyculin A, the phosphorylation of AKT at Ser473, as well as the phosphorylation of its downstream effectors, TSC2 and S6, were maintained in Dock7 KO cells and Dock7 was no longer required to maintain AKT phosphorylation under serum free conditions (Fig. 3h). We further probed potential interactions between AKT and known phosphatases for AKT, PP2AB and PHLPP by PLA, which suggested a significant increase in AKT–PP2AB and AKT–PHLPP interactions in Dock7 KO cells compared to WT cells when cultured in serum-free media, but not when cultured under normal growth conditions (Fig. 3i,j). Interestingly, Dock7 KO appeared to have a more significant impact on AKT interactions with PHLPP, a phosphatase that shows substrate specificity for AKT phosphorylation at Ser473,^73^ during serum starvation compared to PP2AB. These findings indicate that Dock7 preserves AKT activity by protecting AKT from dephosphorylation, thereby sustaining a sufficient level of AKT-mTOR signaling for cancer cells to survive under conditions of serum starvation.

### Both evolutionarily conserved Dock domains, DHR1 and DHR2, contribute to maintaining AKT activation and the transforming potential of Dock7.

**Figure 4.**
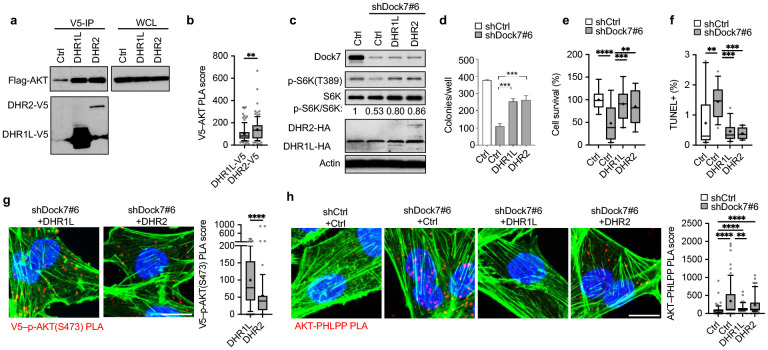
Dock7 DHR1 and DHR2 domains both interact with AKT and provide phenotypic rescue from the cellular loss of Dock7, while DHR1 protects AKT from dephosphorylation. (**a**) Flag-tagged AKT was transiently overexpressed in HEK-293T cells semi-stably expressing either DHR1L-V5 or DHR2-V5. Immunoprecipitation was performed using anti-V5 beads and complexes were resolved on an SDS-PAGE gel for Western analysis. (**b**) PLA of V5–AKT interactions following Dock7 knock-down and DHR1L and DHR2 overexpression in MDA-MB-231 cells grown in serum free medium. (**c, d**) Dock7 was semi-stably knocked down in Hela cells using shRNA and either the DHR domains or an empty vector were expressed using the lentiviral system. Cells were then seeded in either (**c**) 100 mm plates for protein analysis using Western blotting or (**d**) in soft agar suspension and counted two weeks later. (**e**) Cell survival of MDA-MB-231 cells following Dock7 knockdown and DHR1L and DHR2 overexpression after 2 days in serum-free media. (**f**) TUNEL assay of MDA-MB-231 cells following Dock7 knockdown and DHR1L or DHR2 overexpression after overnight serum starvation. (**g**) PLA of interactions between the V5-tag of DHR1L or DHR2 and p-AKT(S473) in MDA-MB-231 cells following Dock7 knockdown and limit domain overexpression. (**h**) PLA of interactions between endogenous AKT and PHLPP following Dock7 knock-down and DHR1L or DHR2 overexpression in MDA-MB-231 cells following overnight serum starvation. Data shown as median ± interquartile range (box), 5–95 (whiskers), and mean (+), or mean ± s.d. *P<0.05, **P<0.001, ***P<0.0001, ns = not significant.

To better understand how Dock7 was interacting with and protecting AKT, we examined the roles of the two conserved Dock7 domains in maintaining AKT phosphorylation at Ser473. For these studies, we used constructs encoding a DHR2 limit domain (DHR2) and a DHR1 domain with a C-terminal 183 amino acid addition of the downstream linker region (DHR1L), which was anticipated to express better than the DHR1 limit domain (DHR1E for DHR1-exact). Co-immunoprecipitates isolated from HEK-293T cells semi-stably expressing V5-tagged DHR1L or DHR2 and ectopically expressing Flag-AKT showed the ability of both DHR1L and DHR2 to interact with AKT (Fig. 4a). Given that significantly less DHR2 was expressed in these cells compared to DHR1L, their comparable effectiveness in co-precipitating with AKT suggests that AKT undergoes a stronger interaction with the DHR2 domain. PLAs also suggested an increased association between DHR2 and endogenous AKT relative to DHR1L during serum starvation, consistent with DHR2 being capable of binding AKT with a higher affinity compared to DHR1L (Fig. 4b).

Given that both DHR1L and DHR2 associated with AKT, we tested the ability of DHR1L and DHR2 to restore the activation of mTOR, as read-out by the phosphorylation of S6K at Thre389, and the transformed phenotype that was lost when Dock7 was knocked down. We found that low levels of the ectopic expression of each Dock7 domain in HeLa cells depleted of endogenous Dock7 could partially restore mTOR activity and the ability of cells to form colonies in soft-agar (Fig. 4c,d), as well as restore their capability to survive serum-deprivation (Fig. 4e) and to be protected from undergoing apoptosis (Fig. 4f). However, while both DHR1L and DHR2 showed an ability to interact with active AKT (Fig. 4a), we found by PLA, that DHR1L expressed in Dock7 knockdown cells showed a greater capability to associate with p-AKT(S473) relative to DHR2 (Fig. 4g). Correspondingly, the expression of DHR1L in Dock7 knockdown cells rescued AKT from interactions with its phosphatase, PHLPP, to a greater extent than did DHR2 (Fig. 4h), thus suggesting the DHR1 domain is responsible for protecting AKT from dephosphorylation.

### Examining the unique role of the DHR1 domain of Dock7.

**Figure 5.**
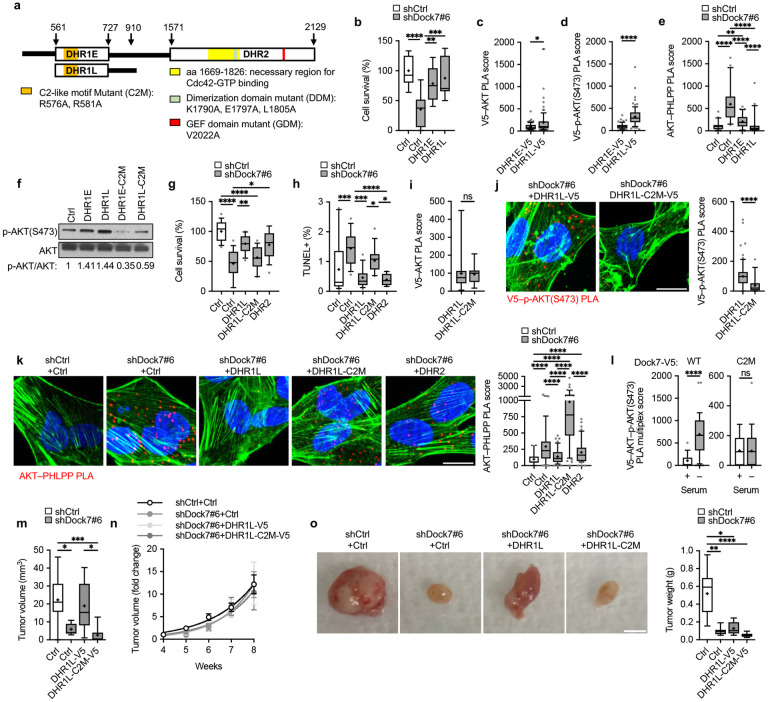
The DHR1 C2-like motif is necessary to protect phosphorylated AKT and sustain AKT and mTOR/S6K activity. (**a**) Schematic of Dock7 and its features. (**b**) Cell survival of MDA-MB-231 cells following Dock7 knockdown and DHR1E and DHR1L overexpression after 2 days in serum-free media. (**c**) PLA of V5–AKT interactions following Dock7 knock-down and DHR1E and DHR1L overexpression in MDA-MB-231 cells grown in complete media or following overnight serum deprivation. (**d**) PLA of V5–p-AKT(S473) interactions following Dock7 knock-down and DHR1E and DHR1L overexpression in MDA-MB-231 cells grown in complete media or following overnight serum deprivation. (**e**) PLA of interactions between endogenous AKT and PHLPP following Dock7 knock-down and DHR1E and DHR1L overexpression in MDA-MB-231 cells following overnight serum starvation. (**f**) Western blot of specified domains and their mutants overexpressed in HeLa WT cells and probing for p-AKT(S473). (**g**) MDA-MB-231 cell survival following Dock7 knockdown and overexpression of DHR constructs. (**h**) TUNEL assay of MDA-MB-231 cells following Dock7 knockdown and DHR1L limit domain overexpression after overnight serum starvation. (**i, j**) PLA of interactions between (**i**) endogenous p-AKT and the V5-tag of each Dock7 construct, and (**j**) endogenous AKT and PHLPP for each construct. (**k**) PLA multiplex for V5–AKT and AKT–p-AKT(S473) PLA in MDA-MB-231 cells transiently overexpressing WT Dock7 and Dock7-C2M. (**l-n**) NSG mouse tumor xenograft of MDA-MB-231 cells following Dock7 knockdown and DHR1L and DHR1L-C2M limit domain overexpression. (**l**) Quantification of tumor volume at 3 weeks post-injection. (**m**) Normalized tumor growth over 4-8 weeks post-injection (fold change normalized to week 4 tumor volumes). Dashed lines show exponential growth. (**n**) Representative images of tumors at with quantification of tumor weight at week 8. Data shown as median ± interquartile range (box), 5–95 (whiskers), and mean (+), or mean ± s.d. *P<0.05, **P<0.001, ***P<0.0001, ns = not significant.

To date, a specific function for the DHR1 domain of Dock7 has not been defined, so we sought to identify its role in the ability of Dock7 to activate mTOR and maintain AKT phosphorylation during serum deprivation. DHR1 contains a putative C2-like motif (Fig. 5a), which has been shown to bind phospholipids and to be important in the subcellular localization of Dock180 and Dock2.^57, 74^ Therefore, we focused our attention to the role of the C2-like motif within the DHR1 domain of Dock7. The sequences of all eleven Dock180-family proteins were aligned and two conserved positive amino acid residues on Dock7 were identified that might be capable of mediating interactions with a negatively charged binding partner. Site-directed mutagenesis was performed to create DHR1 C2 mutants (DHR1E/L-C2M) by substituting alanines for the two conserved arginine residues (R576A, R581A) (Fig. 5a).

We first compared the functionality of the DHR1E and DHR1L constructs by ectopically expressing them in MDA-MB-231 cells. While both constructs displayed efficacy in function, DHR1L was relatively more effective in rescuing cell survival following the knockdown of Dock7, and interacting with AKT and phosphorylated AKT, compared to DHR1E (Fig. 5b-d). Based on PLAs, the expression of DHR1L also appeared to be more effective at reversing the AKT-PHLPP (phosphatase) interactions that occur when Dock7 is knocked down (Fig. 5e). We next assessed what impact mutations to the C2-like motif might have on DHR1E and DHR1L function. The presence of these mutations eliminated the ability of DHR1E and DHR1L to stimulate AKT phosphorylation when expressed in serum starved HeLa cells (Fig. 5f). Survival assays in MDA-MB-231 cells where Dock7 was knocked down, and V5-tagged DHR1L, DHR1L-C2M, DHR2 domains, or vector control were overexpressed, showed that while DHR1L and DHR2 both partially restored cell survival and prevented apoptosis under serum-free conditions, DHR1L-C2M was less effective (Fig. 5g,h). While there was no difference in the number of interactions with AKT, the ectopic expression of DHR1L showed greater potential to interact with phosphorylated AKT relative to DHR1L-C2M by PLA (Fig. 5i,j). Moreover, while the expression of DHR1L and DHR2 were able to reverse the enhanced PLA interactions between AKT and PHLPP that occur upon the knockdown of Dock7, the expression of DHR1L-C2M appeared to further enhance AKT–PHLPP interactions over the knockdown of Dock7 alone (Fig. 5k). We also evaluated AKT interactions with full-length Dock7 and Dock7-C2M constructs in MDA-MB-231 cells. Here, we used the PLA multicolor kit to simultaneously visualize V5–AKT and AKT–p-AKT(S473) interactions and identify Dock7–AKT interactions where AKT was phosphorylated at Ser473. While WT Dock7 showed an increase in the fraction of interactions with phosphorylated AKT when cells were switched to serum-free media, the potential for Dock7-C2M to interact with AKT did not change with the absence of serum (Fig. 5l).

We then examined tumor growth in mouse xenografts where we implanted MDA-MB- 231 WT (vector control) cells, cells where Dock7 had been knocked down, and then Dock7 knockdown cells in which either DHR1L or DHR1L-C2M had been expressed. As expected, Dock7 knockdown cells were not able to form tumors compared to WT MDA-MB-231 cells (Fig. 5m). Interestingly, we found that the expression of DHR1L in Dock7 knockdown MDA-MB-231 breast cancer cells helped the cells to survive the initial implantation and tumor formation during the first 3 weeks of the experiment, whereas cells expressing DHR1L-C2M were completely ineffective (Fig. 5m). We also observed that the control cells containing empty vector, the Dock7 knockdown cells, and the DHR1L and DHR1L-C2M expressing Dock7 knockdown cells, showed no differences in their growth rates after each tumor had formed (Fig. 5n, Supplemental Fig. S4a). This may be due to the ability of tumors to develop a nutrient supply after their initial formation through angiogenesis, thereby becoming less dependent upon the Cdc42-Dock7-mTOR pathway for survival. This observation also supports the notion that Dock7 does not impact cell proliferation. After 8 weeks, the ability of DHR1L to rescue the effects of knocking down Dock7 was reduced (Fig. 5o, Supplemental Fig. S4b).

### The DHR2 domain functions as a dimerization-dependent Cdc42 effector to promote AKT-mTOR activity.

**Figure 6.**
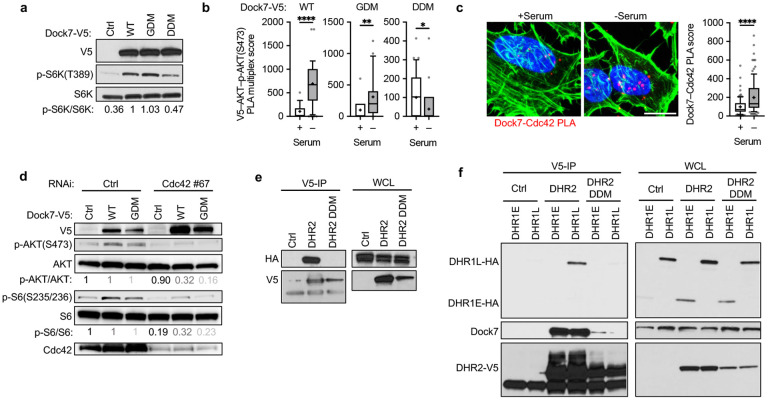
The role of the DHR2 domain in Dock7 function. (**a**) HeLa cells were either mock-transfected or transiently transfected with plasmids to overexpress either V5-tagged WT Dock7 or its GEF-defective mutant (GDM). Cells were serum starved overnight, and then p-S6K was assessed by Western blotting (**b**) PLA multiplex for V5–AKT and AKT–p-AKT(S473) in MDA-MB-231 cells transiently overexpressing WT Dock7, Dock7-GDM, or Dock7-DDM. (**c**) PLA assessing interactions between endogenous Dock7 and Cdc42 in full-serum or following overnight serum starvation in MDA-MB-231 cells. (**d**) Western blot of p-AKT and p-S6K from MDA-MB-231 cells transiently overexpressing Dock7 or Dock7-GDM while Cdc42 was knocked down using siRNA. (**e**) DHR2 was tagged with either HA or V5 and co-expressed in HEK-293T cells. Anti-V5 beads were used to immunoprecipitate V5-tagged DHR2 protein, and Western blots were probed with HA antibody to confirm interactions. (**g**) HA-tagged DHR1L and DHR1E were overexpressed in HEK-293T cells semi-stably expressing either DHR2-V5 or DHR2-DDM-V5 and then collected. Anti-V5 beads were used to immunoprecipitate from the lysate, and Western blot analysis was performed. Data shown as median ± interquartile range (box), 5–95 (whiskers), and mean (+), or mean ± s.d. *P<0.05, **P<0.001, ***P<0.0001, ns = not significant.

The most notable feature of the DHR2 domain of Dock proteins is its ability to promote guanine nucleotide exchange on Cdc42 and/or Rac. In addition to the ability of DHR2 to function as a Cdc42-GEF, we have found it also contains an allosteric binding site for activated GTP-bound Cdc42, located N-terminal to the limit GEF domain^60^ and proximal to a putative dimerization domain (Fig 5a). Therefore, to better understand how the DHR2 domain contributes to the ability of Dock7 to facilitate its atypical activation of AKT-mTOR, we examined the roles of both the GEF and dimerization regions. To this end, we created a Dock7 mutant that contains a substitution of an alanine residue for the conserved, catalytic Val2022 residue within the GEF domain that renders Dock proteins GEF-defective^75^ (GEF-defective mutant, GDM). Based on analogy with other Dock proteins, we also identified 3 amino acid residues which, when mutated to alanines (K1790A, E1797A, L1805A), created a dimerization-defective mutant (DDM) DHR2 domain that was not able to co-immunoprecipitate endogenous Dock7 (Supplemental Fig. S5). Overexpression of Dock7-GDM in cells promoted S6K phosphorylation to a similar extent as WT Dock7 during serum starvation (Fig. 6a), suggesting that under these conditions the GEF activity of Dock7 is dispensable for its ability to activate mTOR. Dock7-DDM, however, was compromised in its ability to signal to mTOR-S6K. These findings were confirmed by PLA. MDA-MB-231 cells were ectopically expressed with WT Dock7-V5, Dock7-GDM-V5, and Dock7-DDM-V5, and then the PLA multicolor kit was used to multiplex PLAs and identify the amount of Dock7-V5 interactions with AKT and phosphorylated AKT. Full-length WT Dock7 and Dock7-GDM showed an increase in phosphorylated AKT interactions following serum withdrawal; however, Dock7-DDM showed a loss of interactions and failed to assemble into an activating complex (Fig. 6b).

It was unexpected that the GEF activity of Dock7 was not required for any Dock7 function we measured during nutrient deprivation, especially since Cdc42 was necessary for Dock7 to promote mTOR/S6K signaling (Fig. 1g). PLA also showed enhanced interactions between Dock7 and Cdc42 during serum starvation, further describing Cdc42 as a member of this stress-activated complex (Fig. 6c). Therefore, we tested if GEF-defective Dock7-GDM also required Cdc42 to promote AKT and S6K stimulation by ectopically expressing either WT Dock7 or Dock7-GDM in cells where Cdc42 had been knocked down and then examining their ability to activate AKT and mTOR. Both WT Dock7 and Dock7-GDM required Cdc42 to stimulate the phosphorylation of AKT and S6 (Fig. 6d).

The observation that Dock7-GDM requires the allosteric binding of Cdc42 to create an active complex, coupled with the inability of a dimerization-defective Dock7 to associate with active AKT, suggested that active Cdc42 binding at the Dock7 allosteric site might lead to a molecular rearrangement of Dock7 dimers to facilitate the assembly of the Dock7-mTOR signaling node and yield a survival response. In addition to finding that DHR2-DDM could not bind to endogenous Dock7 (Supplemental Fig. S5), we further confirmed the specificity of two DHR2 domains to dimerize by co-expressing V5-tagged DHR2 with HA-tagged DHR2 or HA-tagged DHR2-DDM, isolating the V5-tagged DHR2 and observing the inability of the dimerization mutant to co-precipitate with the WT DHR2 (Fig. 6e). What was not obvious, however, was if there were additional intramolecular interactions within Dock7 which could be facilitated by dimer interactions within DHR2 and might provide the potential for greater complex assembly. To examine this possibility, DHR2-V5 or DHR2-DDM-V5 were co-expressed with HA-tagged DHR1E or DHR1L and then the DHR2 constructs were isolated through their V5-tags. Probing by Western blotting confirmed the inability of DHR2-DDM to co-precipitate endogenous Dock7. Interestingly, DHR1L, but not DHR1E, was able to co-precipitate with DHR2 but not the dimerization defective mutant, suggesting that the linker region extension in DHR1L may be important for higher order interactions between DHR domains (Fig. 6f).

### Dock7-mTOR complex assembly.

**Figure 7.**
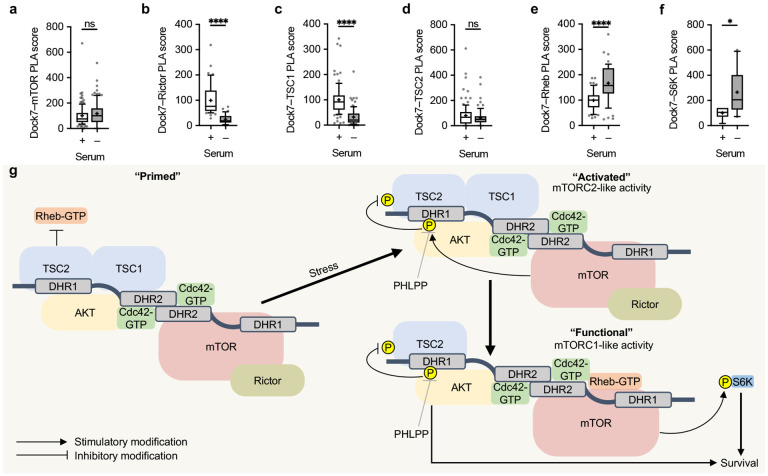
Dock7 complex assembly in response to serum deprivation. (**a-f**) PLA score for interactions between endogenous Dock7 and (**a**) mTOR, (**b**) Rictor, (**c**) TSC2, (**d**) TSC1, (**e**) Rheb, and (**f**) S6K in MDA-MB-231 cells grown in complete media or serum starved overnight. (**g**) Proposed model of Dock7-mediated cellular response to stress. In complete media, the Dock7-mTOR complex is formed and “primed”, and after the stress of serum withdrawal the complex is “activated” showing mTORC2-like activity. Following mTOR-Rictor activation of AKT, Rictor and TSC1 dissociate and the “Functional” Dock7-mTOR complex displays mTORC1-like activity and S6K activation. Data shown as median ± interquartile range (box), 5–95 (whiskers), and mean (+), or mean ± s.d. *P<0.05, **P<0.001, ***P<0.0001, ns = not significant.

We have shown that two of the functionally important binding partners of Dock7, Cdc42 and p-AKT, increase their associations with Dock7 in cancer cells upon the serum withdrawal (Fig. 6c and Fig. 3d, respectively), while pan-AKT–Dock7 interactions appear stable in the presence and absence of serum (Fig. 3c). To further examine the relationships between the other Dock7-associated components and Dock7, we continued to utilize PLAs in MDA-MB-231 cells cultured in the presence or absence of serum. PLA showed that endogenous Dock7 was in sufficient proximity to interact with mTOR both in serum-treated and serum-depleted cells; whereas, Dock7–Rictor interactions decreased under conditions of serum starvation, suggesting a transient mTORC2 activity capable of phosphorylating AKT within the complex (Fig. 7a,b). PLAs also indicated that Dock7–TSC2 interactions were serum independent, but that Dock7–TSC1 interactions were decreased upon serum depletion (Fig. 7c-d), consistent with reports that TSC1 dissociates from the TSC1/2 complex following an inactivating phosphorylation on TSC2 by AKT.^24-27^ Importantly, Rheb and S6K, an mTORC1 activator and effector, both showed enhanced engagement with Dock7 when cells were deprived of serum (Fig. 7e,f). Collectively, this series of experiments suggest a “core” Dock7 complex comprising Dock7, AKT, mTOR and TSC2 which is resistant to fluctuations in cellular nutrient status. Limiting resources result in a molecular rearrangement of Dock7 interacting proteins favoring a complex capable of both sustaining AKT activity and activating a Dock7-dependent mTORC1-like activity to provide cancer cells with survival benefits.

These results, when taken together with those described in the preceding sections, lead us to propose the working model depicted in Fig. 7g and further discussed below. We suggest that Cdc42-GTP dependent interactions within DHR2 can stabilize Dock7 dimers in a structural orientation that enables Dock7 to serve as a signaling scaffold to assemble a complex that initially forms when cells are exposed to serum (depicted as “primed”). This state would favor an activatable mTORC2 activity and be inhibitory toward a functional mTORC1-like activity. Stress signals would begin a cascade of events leading to an “activated” state, first with the phosphorylation of AKT at S473 by Rictor associated-mTOR, followed by the phosphorylation of TSC2 at T1462 by AKT. The Dock7 complex would become “functional” following the subsequent dissociation of Rictor and TSC1, which would generate conditions favorable for a Dock7-associated mTORC1-like activity by disabling the GAP activity of TSC2. Pools of Rheb-GTP would become available to activate the Dock7-associated mTOR allowing for its subsequent phosphorylation of S6K. This “functional” Dock7 complex becomes a survival signaling node through its ability to preserve the activity of AKT under adverse conditions, ultimately propagating survival signals through AKT effectors, including the Dock7-associated mTORC1-like activity described in this study.

## Discussion

Our discovery of a unique Cdc42- and Dock7-dependent mTOR signaling complex that is required for the malignant phenotypes and survival of cancer cells during stresses like those observed within the tumor microenvironment emerged from efforts to address a long-standing question of how Cdc42 promotes the activation of mTOR in different cellular contexts. Early work showed that both the Cdc42 and Rac1 GTPases were capable of stimulating S6K phosphorylation, a well-known downstream target of mTOR, in growth factor-stimulated cells.^40^ Studies from our laboratory then uncovered additional growth factor-dependent signaling connections between Cdc42 and mTOR that play important roles in cap-dependent mRNA splicing,^41^ and the ability of multi-potent teratoma cells and pluripotent embryonic stem cells to transition to neuro-progenitor/neural stem cells^44^. However, here we have elucidated a Cdc42-promoted signaling complex that is able to provide a sufficient stimulation of mTOR activity in the absence of growth factors, such that cancer cells can survive when confronted with severe stresses such as serum deprivation.

An important clue regarding how Cdc42 directs the formation of this novel mTOR signaling complex came from proteomics studies that suggested Dock7, a Cdc42-GEF and binding partner for activated GTP-bound Cdc42,^60^ interacts with the TSC1/TSC2 complex,^61^ the GTPase-activating protein and negative regulator of the small GTPase Rheb. We found that in addition to TSC1/TSC2, Dock7 can interact with mTOR, Rheb, and AKT, based on its ability to be co-immunoprecipitated with these proteins and that they are in sufficient proximity in cells to form complexes based on PLA. Moreover, we showed that the ectopic expression of Dock7 gives rise to a Rapamycin-sensitive and Rheb-dependent activation of mTOR/S6K during serum deprivation through its ability to preserve a basal level of AKT activity. Inhibition of this basal AKT activity completely prevents cancer cells from growing in soft agar. However, the knock-down of S6K reduced the ability of cancer cells to grow in soft agar by half, indicating S6K activity is an important, but not exclusive AKT signaling effector in the Dock7 pro-survival response, as AKT directs additional downstream signals that block apoptosis. It will be important to understand what these downstream signals are, and further proteomic and phospho-proteomic studies will be necessary to define the global changes that occur downstream of the Cdc42-Dock7-mTOR signaling axis during stress. Such studies will help to better distinguish the functions and molecular mechanisms of this distinct signaling node from the previously described growth factor-dependent AKT and mTOR pathways.

Although our findings collectively point to the ability of Dock7 to regulate mTORC1, we find that Dock7-dependent stimulation of mTOR/S6K is Raptor-independent and does not appear to function at the lysosome. It has been well established that mTORC1 is activated at the lysosome in response to mitogenic signaling and amino acids, which is facilitated by Raptor,^17-21^ and is inhibited when growth conditions are limited due to the phosphorylation of Raptor by AMPK.^76^ However, there have been other reports of Rapamycin-sensitive, Raptor-independent mTORC1-like activities.^77, 78^ Previous studies suggest that Raptor-independent S6K recruitment occurs through interactions with the FKBP-Rapamycin binding (FRB) domain of mTOR, which places S6K in close proximity to the active kinase domain.^22, 79^ Substrate recruitment by the FRB domain allows for Rapamycin sensitivity to be maintained in the absence of Raptor, as Rapamycin forms an inhibitory complex with FKBP12 and subsequently binds to the FRB domain of mTOR.^80^ The mTORC2 component, Rictor, also partially obscures the FRB domain of mTOR, which gives rise to the mTORC1 specificity of Rapamycin.^81^ Therefore, our finding that Dock7 stimulation of S6K/mTOR requires Rictor and is Rapamycin sensitive indicates that there are both Rictor-dependent (mTORC2-like) and Rictor-independent (mTORC1-like) mTOR functions occurring within the Dock7 signaling complex. In contrast to canonical mTORC1 signaling, Raptor-independent mTOR complexes may provide a mechanism to generate targeted and context-dependent mTOR/SK6 activity when canonical mTORC1 activity is disabled.

This Dock7-dependent AKT activity that is observed upon the removal of serum from culture conditions may have previously been considered “background”, but we now show that this activity is critically important for resistance to anoikis and apoptosis in cancer cells. In response to nutrient and growth factor stimulation, the canonical pathway of AKT activation by RTKs or GPCRs leads to its recruitment to the plasma membrane, where it binds with PIP_3_ through its PH domain, promoting AKT phosphorylation. Upon activation, AKT can then phosphorylate and inhibit TSC2, thus activating Rheb and mTORC1, and results in mTOR localizing to the lysosome, where it is activated.^82^ It was initially thought that after phosphorylation AKT dissociates from the plasma membrane, diffusing throughout the cytosol to phosphorylate its target proteins;^83, 84^ however, AKT is rapidly de-phosphorylated after release from a membrane as its unbound PH domain allosterically blocks accessibility to binding and regulatory sites, providing robust autoinhibition.^85, 86^ While membrane and ATP binding are primarily responsible for protecting AKT from dephosphorylation by phosphatases,^86-89^ other proteins have been shown to bind AKT and prevent phosphatase-mediated dephosphorylation.^90, 91^ Dock6, another Dock-C member of the Dock180 family, has also been reported to interact with the kinase domain of AKT through its DHR1 domain.^92^ However, Dock7 binding to the phosphorylated AKT appears to occur through a distinct mechanism specific to the C2-like motif of its DHR1 domain, as C2 and C2-like motifs have not previously been implicated in binding to phospho-proteins. We show that the C2-like motif bound phosphorylated AKT and protected it from dephosphorylation to preserve a pool of active AKT during serum deprivation, highlighting a new model for how cancer cells can sustain the activity of AKT and mTOR/S6K during environmental stress.

Dock7 has been mainly studied in brain development where it regulates neuronal polarity and Schwann cell migration^93, 94^ and initial studies suggest a similar classical role for Cdc42/Rac activity in cancer.^95-97^ However, we find that GEF activity is dispensable for Dock7 stimulation of AKT and mTOR/S6K and that Dock7 acts as a scaffold. A unique aspect of this signaling complex is that the lesser studied DHR1 domain of Dock7 binds to phosphorylated AKT and helps to maintain a basal level of AKT activation. Very little is known about the DHR1 domains of the Dock family of atypical GEFs relative to the DHR2 domains, which confer the better-studied GEF activity. For two members of the family, Dock180 and Dock2, the DHR1 domain is known to be a membrane phospholipid motif that mediates the localization of these proteins.^57, 58^ We were initially surprised to find that DHR1 rescued anchorage-independent growth, cell survival, and signaling to S6K in cells depleted of Dock7, suggesting that the DHR1 domain provides an essential function for the ability of Dock7 to enable cancer cells to survive certain forms of cellular stress. The finding that DHR1 promotes AKT activity by preserving its activated state explains how DHR1 can propagate signaling and help to restore transformed phenotypes that are lost when depleting cells of endogenous Dock7. However, it is likely that the DHR1L construct only partially and not fully recapitulates the actions of Dock7 in transformation assays and xenograft experiments due to the role that the full-length Dock7 protein plays as a signaling scaffold in the Cdc42-dependent activation of mTOR.

In conclusion, our findings from immunoprecipitation experiments and PLA analysis suggest a model in which Cdc42, together with its signaling partner Dock7, directs the assembly of a multi-protein complex that maintains the activated state of AKT and enables a sufficient stimulation of mTOR/S6K activity for cancer cells to survive stresses such as serum deprivation. Like other Dock family members, Dock7 exists as a dimer, with the allosteric binding site for Cdc42 being proximal to the dimerization domain. Therefore, the binding of Cdc42 at this site might promote the Dock7 dimer to transition from an autoinhibited state to an active/open conformation which would then expose the DHR1 C2-like motif, enabling it to engage p-AKT(S473) and protect it from dephosphorylation by phosphatases. The DHR2 domain of Dock7 may play a role in stably engaging AKT, allowing phospho-Ser473 to become proximal to the C2-like motif of DHR1 thereby facilitating interactions with AKT. Other regions on Dock7 likely serve scaffolding functions to enhance the interactions with downstream effectors such as the TSC complex, and to assemble mTOR and its activator Rheb to generate a targeted, stress-dependent mTOR/S6K activity. Based on our estimates of the molecular mass of the complex from native-PAGE, we show only a single mTOR and not an mTOR dimer being present. Thus, we suggest that Rictor-associated mTOR, which is initially present in the “primed” Dock7-mTOR complex, as indicated by its co-immunoprecipitation with Dock7 and by PLA, becomes activated upon cellular stress (i.e., within the “functional and active” complex) and phosphorylates AKT at Ser473. At this point Rictor dissociates based on PLA analysis, which would terminate mTORC2-like activity and AKT phosphorylation.^98^ However, the sustained activation of AKT by Dock7 and phosphorylation of TSC2 by AKT within the active complex results in the dissociation of its partner subunit TSC1, again as indicated by PLA, which would enable Rheb to maintain its GTP-bound state to re-activate mTOR. The model presented may present limit complexes, as other components such as additional mTORC2 regulatory factors aside from Rictor may be present. It also is still unclear how Cdc42 is initially activated to start this signaling process, since we found that a GEF-defective mutation in the DHR2 domain of Dock7 did not prevent it from signaling to mTOR. A likely explanation is that there exists a pool of pre-formed Cdc42-GTP in growing cells that is maintained in an active state during cellular stress by binding to the allosteric site on Dock7. It has been shown that signaling partners of GTP-bound Cdc42 can block GTP hydrolysis, helping Cdc42 remain in an activated state.^99, 100^ It will be necessary, moving forward, to better understand how the spatiotemporal dynamics of identified players within the cell given the large molecular sizes of the proteins involved in this complex, as well as elucidate the structural features of the different protein-protein interactions that comprise this signaling node.

## Materials and Methods

### Cell lines, cell culture, and reagents.

HEK-293T and all cancer cells were obtained from American Type Cell Culture Collection (ATCC), except TSE breast cancer cells (kindly supplied by Dr. Steven Abcouwer, University of Michigan), and the MDA-MB-231 cells metastasized to brain (kindly supplied by Dr. Joan Massagué, MSKCC^101^). Cancer cells were maintained at 37°C, 5% CO_2_ in RPMI 1640 (Thermo Fisher) supplemented with 10% fetal bovine serum (FBS; Thermo Fisher), HEK-293T cells were maintained at 37°C, 5% CO_2_ in DMEM supplemented with 10% FBS. For growth factor stimulation, 7x10^5^ cells were seeded in 100 mm dishes (Corning), serum-starved for 20-24 h, then stimulated with Heregulin β (HRG), EGF domain, residues 178-241 (Sigma-Aldrich) at the concentration and times indicated, followed by cell lysis. All cell lines were tested and found negative for mycoplasma contamination.

### DNA constructs, siRNA, and shRNA.

Rac1, Cdc42, Rheb, Dock7, wild-type and point mutation constructs used for transient transfections were cloned in our laboratory into pcDNA3.1 (Thermo Fisher). TSC1, TSC2, and mTOR DNA constructs were obtained from Addgene (plasmid #12133, #14129, #1861)^24, 26, 30^. Dock7 lentiviral constructs, Dock7 truncations, Rac1, Cdc42, Rheb, and YFP used for transformation assays were cloned into pSIN-EF2. Constructs of Dock7 contained the following amino acids: DHR1E (561-727), DHR1L (561-910), DHR2 (1571-2129). GST-Rheb was cloned into pGEX (GE Healthcare Life Sciences). Silencer Select siRNAs of Rheb (s12019, s12020, s12021), Rac1 (s11711, s11713), and Cdc42 (s2765, s2766, s2767) were purchased from Thermo Fisher. Dock7 shRNAs and negative control were purchased from Sigma (shRNA1: TRCN0000377466, shRNA3: TRCN0000365143, shRNA6: TRCN0000365145) and virus particles were generated according to manufacturer’s protocol.

### Transfection.

7x10^5^ cells were seeded in 100 mm dishes (Corning) then transfected with 4 μg DNA using Lipofectamine and Plus Reagent (Thermo Fisher) according to the manufacturer’s protocol. Cells recovered in complete medium for 3 h followed by serum starvation for 20- 24 h. For knock-down experiments, cells were seeded and transfected with 2.5 nM siRNA the next day using Lipofectamine2000 (Thermo Fisher) following the manufacturer’s protocol. For rescue experiments, after siRNA transfection cells were then split onto 60 mm dishes at 2.5x10^5^ cells/dish, allowed to recover overnight, and transfected with 1 μg of DNA using Lipofectamine and Plus Reagent. For HEK-293T cells, 3x10^6^ cells were seeded in 100 mm dishes, cultured for 24 h, and transfected with 4 μg DNA the next day using Lipofectamine and Plus Reagent following the manufacturer’s protocol.

### Lentiviral transduction.

To generate lentivirus, HEK-293T cells were cultured for 24 h in 100 mm dishes to 80% confluency and transfected with constructs. 6 μg of construct DNA (pSIN for overexpression or pLKO for shRNA), 4 μg pCMV, and 2 μg pMD2.G were added to 800 μl serum-free DMEM and mixed with 30 μl PEI. DNA/PEI/DMEM solution was incubated for 15 min at room temperature, then added to cells in 12 ml of fresh media and cultured overnight. Media was then changed to 13 ml of complete media, cells were cultured for 24 h, and spent media was collected. To harvest virus, spent media was centrifuged at 1000 rpm for 10 min to remove cell debris, sterile filtered using a 0.45 μm syringe filter. 13 ml media was replaced and after 24 h virus was collected again. Collected virus was combined, mixed, pipetted into 3 ml aliquots, and stored at −80°C. For lentiviral transduction, 7x10^5^ target cells were seeded in 100 mm plates, cultured overnight, lentivirus with polybrene (1:1000) in complete media was added. Cells were cultured overnight, washed with 1x PBS, cultured for 48 h in complete media, then passaged. Cells expressing constructs were selected with 2 μg/ml puromycin for 3-5 days to create semi-stable lines and maintained in complete media supplemented with 1 μg/ml puromycin.

### CRISPR-Cas9 knock-out.

Dock7 was genetically ablated using CRISPR-Cas9 knock-out plasmid and HDR plasmid transfection. Cells were seeded in 6-well plates at 2.5x10^5^ cells/well in antibiotic-free complete media, allowed to recover overnight, then treated. Solutions of 1 μg CRISPR (Santa Cruz Biotechnology, sc-404461) and DOCK7 HDR (h) (Santa Cruz Biotechnology, sc-404461-HDR) plasmid DNA were prepared and mixed, then added to 150 μl Plasmid Transfection Medium (Santa Cruz Biotechnology, sc-108062). A separate solution of 5 μl UltraCruz Transfection Reagent (Santa Cruz Biotechnology, sc-395739) in 150 μl Plasmid Transfection Medium was also prepared and mixed, then both solutions were incubated for 5 min. Solutions were mixed, incubated for 15 min, and added to cells. Media was changed after 24 h, then after 48 h selection was performed with 1.75 μg/ml puromycin.

### Immunoblot analysis.

Cells were lysed with cell lysis buffer (50 mM Hepes pH 8.0, 150 mM NaCl, 1 mM MgCl_2_, 25 mM NaF, 1 mM Na_3_VO_4_, 50 mM β-glycerophosphate, 10 μg/ml Leupeptin, 10 μg/ml Aprotinin, and 1% Triton X-100). The lysates were resolved by SDS-PAGE (4-20% Tris-Glycine gels, Thermo Fisher), and then the proteins were transferred to polyvinylidene fluoride (PVDF) membranes (PerkinElmer). The membranes were incubated with the indicated primary antibodies diluted in 20 mM Tris pH 7.4, 150 mM NaCl, and 0.05% Tween-20. Primary antibodies were detected with horseradish peroxidase-conjugated secondary antibodies (Cell Signaling Technology) followed by exposure to ECL reagent (PerkinElmer).

### Immunoprecipitation.

HEK-293T cells were lysed with lysis buffer (50 mM Hepes pH 8.0, 150 mM NaCl, 1 mM MgCl_2_, 25 mM NaF, 1 mM Na_3_VO_4_, 50 mM β-glycerophosphate, 10 μg/ml Leupeptin, 10 μg/ml Aprotinin, 0.3% CHAPS). Lysates were pre-cleared with BSA-coated Protein G beads (Thermo Fisher) on a rotator at 4°C for 15 min. The supernatant was collected and added with anti-Myc (Covance), HA (Covance), or Flag (Sigma-Aldrich) antibody for 2 h, then BSA-coated Protein G beads were added and incubated for 1 h at 4°C. Immunoprecipitates were washed 3x with lysis buffer followed by the addition of 2x Laemmli sample buffer. For nucleotide binding to HA-tagged Cdc42, Rac1, and Rheb, cells were transfected and lysed as described above. Cell lysates were then treated with 10 mM EDTA, 10 mM EDTA + 1 mM GDP, or 10 mM EDTA + 100 μM GTPγS at room temperature for 15 min. 50 mM MgCl_2_ was then added to samples containing nucleotides followed by immunoprecipitation as described above.

### Blue Native PAGE.

Blue Native PAGE was performed using lysates prepared from growing HEK-293T cells according to the manufacturer’s protocol (Thermo Fisher). 1×10^7^ cells were collected and lysed using 1x Native PAGE lysis buffer including protease inhibitor cocktail and 1% digitonin. Multiple lanes of the same lysates were then run on one 3-12% Bis-Tris gel followed by denaturing treatment and transferred onto PVDF membrane. Each individual strips of lysates were then blotted for mTOR, Dock7, TSC1, and TSC2. All antibodies were purchased from Cell Signaling Technology. Blots were developed as described above followed by processing and compilation using ImageJ^85^.

### Nucleotide-dependent GST fusion protein pull-down.

GST and GST-Rheb were expressed in BL21 cells and purified by affinity chromatography using Glutathione Sepharose High Performance beads (GE Healthcare Life Sciences) according to the manufacturer’s protocol. GST and GST-Rheb were stored on the glutathione beads with 30% glycerol at −20°C until use. HEK-293T cells were infected with lentivirus containing constructs overexpressing either DHR1-V5 or DHR2-V5. 48 h after infection, cells were selected with 2 μg/mL puromycin for 48 h to create semi-stable cell lines of DHR1-V5 and DHR2-V5. Cells were maintained in 1 μg/mL puromycin after selection. Whole cell lysates of DHR1-V5 and DHR2-V5 were lysed with cell lysis buffer (50 mM Hepes pH 8.0, 150 mM NaCl, 1 mM MgCl_2_, 25 mM NaF, 1 mM Na_3_VO_4_, 50 mM β-glycerophosphate, 1 mM DTT, 10 μg/ml Leupeptin, 10 μg/ml Aprotinin, 1% Triton X-100). Lysates were pre-cleared using GST beads. GST-Rheb was treated with 10 mM EDTA, 10 mM EDTA + 1 mM GDP, or 10 mM EDTA + 100 μM GTPγS at room temperature for 15 min followed by the addition of 50 mM MgCl_2_ for the samples containing nucleotides. GST controls were either treated with EDTA or non-nucleotide loaded (no EDTA). Nucleotide-loaded or nucleotide-free GST/GST-Rheb were added to the pre-cleared lysates of DHR1-V5 or DHR2-V5. Pull-downs were done in the lysis buffer ± EDTA at 4 °C for 2 h. Pulled-down proteins were washed 3x with the lysis buffer ± EDTA, 2x Laemmli sample buffer was added, and analyzed on 12% Tris-Glycine gels.

### Soft agar colony formation.

Cells in complete media containing 0.3% agarose were seeded at 0.8-1×10^4^ cells/well onto a layer of 0.6% agarose with complete media in 6-well plates. SK-BR-3, MCF7, and MDA-MB-231 cells were seeded at 5×10^3^, 1×10^4^ and 2×10^4^ cells/well, respectively, and cultures were fed every 3-4 days with complete medium containing 0.3% agar. After 14-21 days, 1 mg/ml NBT in 1x PBS was added, cultured overnight, and samples were imaged.

### Cell survival assay.

Cells were seeded in 48-well plates at 16,000 cells/well and cultured overnight in complete media. The next day, cells were washed with 1x PBS and media was changed to serum-free media, with fresh media replaced every day. After two days cells were then trypsinized and counted.

### EdU proliferation assay.

DNA synthesis was directly measured in live cells with the EdU Staining Proliferation kit iFluor 488 (Abcam, ab219801) following the manufacturer’s protocol. Cells were seeded in 6-well plates with no. 1.5 22x22 mm coverslips (Electron Microscopy Sciences, 72204-01) at 1.5x10^5^ cells/well, cultured for 48 h, then washed with 1x PBS, and treated with complete or serum-free media. After a 20-24 h incubation, cells were incubated with 10 μm EdU solution for 4 h, fixed in 4% paraformaldehyde (PFA; Thermo Fisher, J19943.K2) for 10 min, permeabilized, and labeled using kit components. Coverslips were mounted using Vectashield with DAPI (Vector, H-1200) microscope slides and sealed. The ImageJ Particle Analyzer plugin was used to calculate proliferation as the fraction of EdU positive cells.

### Cancer spheroid invasion.

MDA-MB-231 cancer cell spheroids were generated as previous described^102^. Spheroids were encapsulated in 3 mg/ml collagen (Corning) and then spheroid invasion was monitored over 24-48 h post-embedding.

### TUNEL assay.

Cells were seeded at 1.5x10^5^ cells/well in 6-well plates with no. 1.5 22x22 mm coverslips, cultured for 48 h in complete media, then washed with 1x PBS, and then treated with complete or serum-free media for 18-24 h. After the incubation, media was removed and cells were washed 3x with 1x PBS, fixed in 4% PFA (Thermo Fisher, J19943.K2) for 10 min, washed 3x with 1x PBS, and stored in 1x PBS at 4°C until the In Situ Cell Death Detection Kit, Fluorescence (Roche, 11684795910) was used according to the manufacturer’s protocol. Analysis was performed in ImageJ using the Particle Analyzer plugin.

### Immunofluorescence staining.

Cells were seeded at 1.5x10^5^ cells/well in 6-well plates with no. 1.5 22x22 mm coverslips, cultured for 48 h, washed with 1x PBS, and treated with complete or serum-free media for 18-24 h. Samples were then washed with 1x PBS, fixed in 4% PFA for 10 min, and washed 3x with 1x PBS. Samples were permeabilized with a 15 min wash in 0.1% Triton X-100, washed 3x with 0.02% Tween20/1x PBS for 15 min each, then blocked in 0.02% Tween20/3% BSA/10% FBS for 4 h. After blocking, samples were incubated in primary antibodies for Dock7 (1:200; Santa Cruz Biotechnology, sc-398888) and LAMP1 (1:200, Cell Signaling Technology, 9091S) overnight at 4°C. Samples were next washed 3x with 0.02% Tween20/1x PBS for 15 min each and incubated with secondary antibodies and counterstains for 2 h at room temp. Following a 3x wash with 0.02% Tween20/1x PBS for 15 min each, and coverslips were mounted on a microscope slide using Vectashield mounting media with DAPI and sealed.

### Proximity ligation assay (PLA).

Cells were seeded at 1.5x10^5^ cells/well in 6-well plates with no. 1.5 22x22 mm coverslips, cultured for 48 h, washed with 1x PBS, and treated with complete or serum-free media for 18-24 h. After incubation, cells were washed 3x with 1x PBS, fixed in 4% PFA (Thermo Fisher, J19943.K2) for 10 min, washed 3x with 1x PBS, and stored in 1x PBS at 4°C until use. Samples were permeabilized with 0.1% Triton X-100 (Millipore, 1.08603.1000) for 15 min and washed 3x for 15 min with 0.02% Tween20 (Sigma-Aldrich, P1379) in 1x PBS. PLA was then performed using Duolink In Situ PLA Probe Anti-Mouse PLUS (Sigma-Aldrich, DUO92001), Duolink In Situ PLA Probe Anti-Rabbit Minus (Sigma-Aldrich, DUO92005), and In Situ Detection Reagents Red (Sigma-Aldrich, DUO92008) following the Duolink PLA Fluorescence protocol. Target proteins were detected using primary antibodies for Dock7 (1:100; Santa Cruz Biotechnology, sc-398888), Dock7 (1:100; Abcam, ab118790), mTOR (1:100; Cell Signaling Technology, 2983S), TSC1 (1:100, Cell Signaling Technology, 6935S), TSC2 (1:100; Cell Signaling Technology, 4308S), Rheb (1:100, Cell Signaling Technology, 13879S), Raptor (1:100; Cell Signaling Technology, 2290S), Rictor (1:100; Cell Signaling Technology, 2114S), LAMP1 (1:100, Cell Signaling Technology, 9091S), AKT (1:100; Cell Signaling Technology, 9272S), PHLPP (1:50, Proteintech, 22789-1-AP), and PP2AB (1:50, Cell Signaling Technology, 4953T), p-AKT(S473) (1:50, Cell Signaling Technology, 4051S), p-TSC2(T1462) (1:50, Cell Signaling Technology, 3617S), V5-tag (1:100, Cell Signaling Technology, 13202s), and Cdc42 (1:100, Cell Signaling Technology, 2466S). PLA multicolor was used following the Duolink Multicolor Detection protocol. Primary antibodies for V5-tag (Invitrogen, MA5-15243) and pan AKT (R&D Systems, MAB2055) conjugated to Duolink PLA Multicolor Probemaker Kit Green (Sigma Aldrich, DUO96020), while pan AKT (R&D Systems, MAB2055) and p-AKT S473 (R&D Systems, AF887) were conjugated with the Probemaker Kit Red (Sigma-Aldrich, DUO96010). Duolink PLA Multicolor Reagent Pack (Sigma-Aldrich, DUO96000) was used for amplification and detection. In Situ Wash Buffers Fluorescence (Sigma-Aldrich, DUO82049) were used for all wash steps in PLA procedures. Analysis was performed in ImageJ, and PLA puncta were counted using the Find Maxima plugin. PLA scores were determined by normalizing the PLA spot count per cell to the average number of PLA spots in control cells, which was set to 100.

### Fluorescence microscopy.

Imaging was performed on a Keyence BZ-X810 inverted fluorescence phase contrast microscope using a Plan Apochromat 60x/1.4 NA oil immersion objective (BZ-PA60, Keyence Corp) and ET DAPI (ex. 395/25 em. 460/50; Chroma, 4900-UFI) ET EGFP (ex. 470/40 em. 525/50; Chroma, 49002-UFI), and ET mCH/TR (ex. 560/40 em. 630/75; Chroma, 49008-UFI) filters was used for fluorescence imaging. PLA Images were taken with optical sectioning (1D, width=10) and z-stacks (0.5 m pitch) taken through the height of the cells.

### Isolation of Mouse Embryo Fibroblasts (MEF).

Pregnant mice were euthanized as approved by AICUC 14 days after appearance of copulation plug. The uterus was removed, and embryos were extracted and placed in 60 mm tissue culture dishes. The head was removed and used for genotyping, and internal organs were discarded. Each embryo was washed with HBSS, without calcium nor magnesium (Thermo Fisher #14170120) twice, and then placed in 0.25% trypsin-EDTA (Corning, #25053CI). Embryonic tissue was minced into small pieces, and incubated at 5% CO_2_, 37°C for 5 min. The mixture was pipetted up and down and placed back in the incubator for 10 mins, trypsin was then deactivated with 15 mL DMEM+10% FBS and transferred to a 50 mL tube where it rested for 10 min. Single cells and the cell cluster supernatant were transferred to a 100 mm plate, cells attached overnight, and media was changed to DMEM+10% FBS after 24 h. Cells were used 48 h later.

### Tumor xenograft.

The different conditions of MDA-MB-231 breast cancer cells were pre-prepared and 3x10^6^ cells were injected subcutaneously into each flank of female NSG mice, aged 8 weeks. Tumor size was measured using calipers every 7 days, and tumor volume was calculated using the formula 0.5 x (length x width^2^). Tumors from NSG mice were harvested 56 days after the xenograft inoculation. All mice experiments were carried out according to the protocols (#2003-0097) approved by the Center for Animal Resources and Education (CARE) at Cornell University.

### TCGA Data.

The Cancer Genome Atlas (TCGA) Breast Cancer (BRCA) data set was assessed and analyzed through UCSC Xena browser 87.

### Statistical analysis.

Statistical analysis was performed using GraphPad Prism 9.0. Normality in was tested with the D’Agostino-Pearson omnibus normality test. Data with a Gaussian distribution were compared using a two-tailed Student’s t-test for two groups and one-way ANOVA with Dunn’s post-hoc analysis for multiple groups. Non-parametric tests were used to compare data with a non-normal distribution and a two-tailed Mann-Whitney test was used for two groups, while one-way Kruskal-Wallis test was used for multiple groups. No statistical method was used to determine sample size. All experiments were reproduced at least three independent times.

## Supplementary Material

Supplement 1
