# Supplementary material for: A multiprotein signaling complex sustains AKT and mTOR/S6K activity necessary for the survival of cancer cells undergoing stress
Source: bioRxiv. 2024 Oct 1:2023.01.03.522657. Originally published 2023 Jan 3. Preprint. [Version 3] doi: 10.1101/2023.01.03.522657 (PMC9881951; doi:10.1101/2023.01.03.522657)
Supplement: Supplement 1 [file media-1.pdf]

Supplementary figures and tables

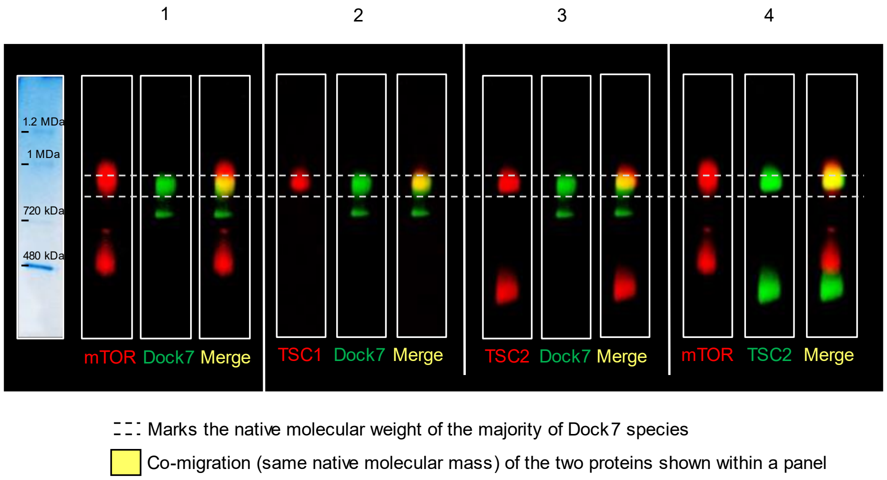

Figure S1. Dock7 co-migrates with mTOR and its main negative regulator, the TSC1/2 complex. HEK-293 cells grown in complete medium were lysed and native complexes were separated using Blue Native-PAGE (BN-PAGE). Each lane was stained by the specified antibodies.

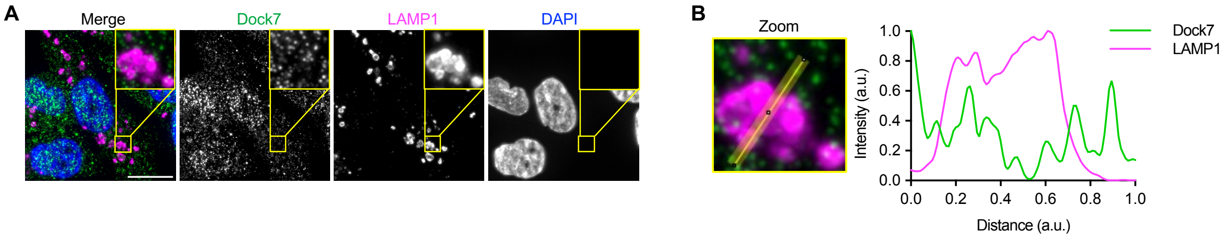

Figure S2. Dock7 does not colocalize with lysosomal-marker LAMP1. (**A**) Immunofluorescence images of Dock7 and LAMP1 in MDA-MB-231 cells in serum-free media. Insert show boxed region of interest (ROI). (**B**) Zoomed ROI and line-scan of Dock7 and LAMP1 fluorescence intensity. Scale bar = 15 µm.

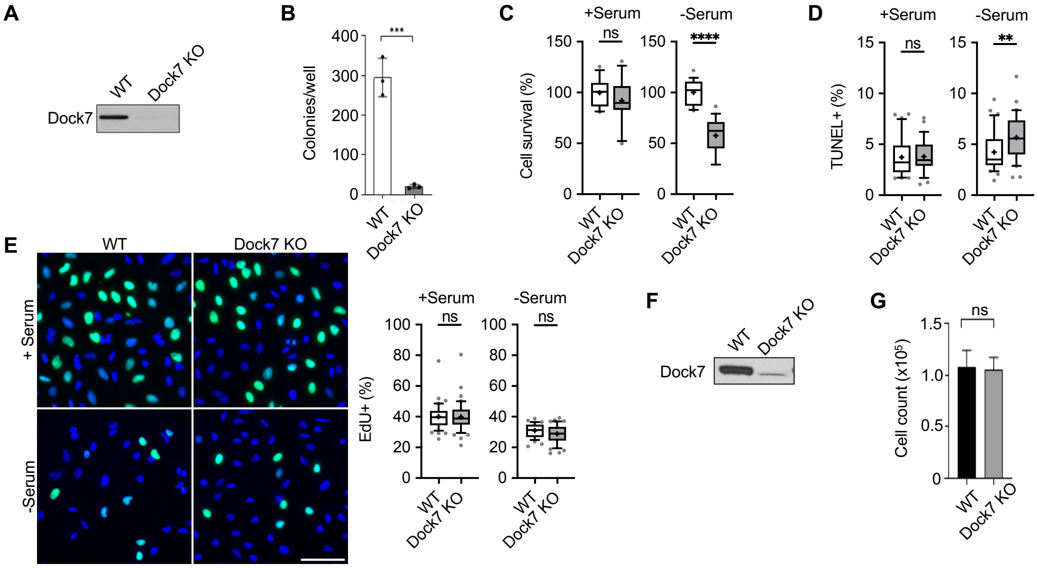

Figure S3. HeLa Dock7 knock-out cell line. (**A**) Western blot of Dock7 expression in HeLa WT and Dock7 KO cell lines. (**B**) Soft agar colony formation of HeLa WT and Dock7 KO cells. (**C**) Cell survival of HeLa WT and Dock7 KO cells in serum-free media after 2 days. (**D**) TUNEL assay of HeLa WT and Dock7 KO cells cultured in complete media or serum-starved overnight. (**E**) EdU assay to determine cell proliferation of HeLa WT and Dock7 KO cells performed in complete media or after overnight serum starvation. (**F**)Western blot of Dock7 protein expression of Dock7 KO MEF cell line. (**G**) Cell survival of WT and Dock7 KO MEF cells cultured in serum-free media for 2 days. Data shown as median ± interquartile range (box), 5th–95th percentiles (whiskers), and mean (+), or mean ± s.d. **P<0.01, ****P<0.0001, ns = not significant. Scale bar = 100 μm.

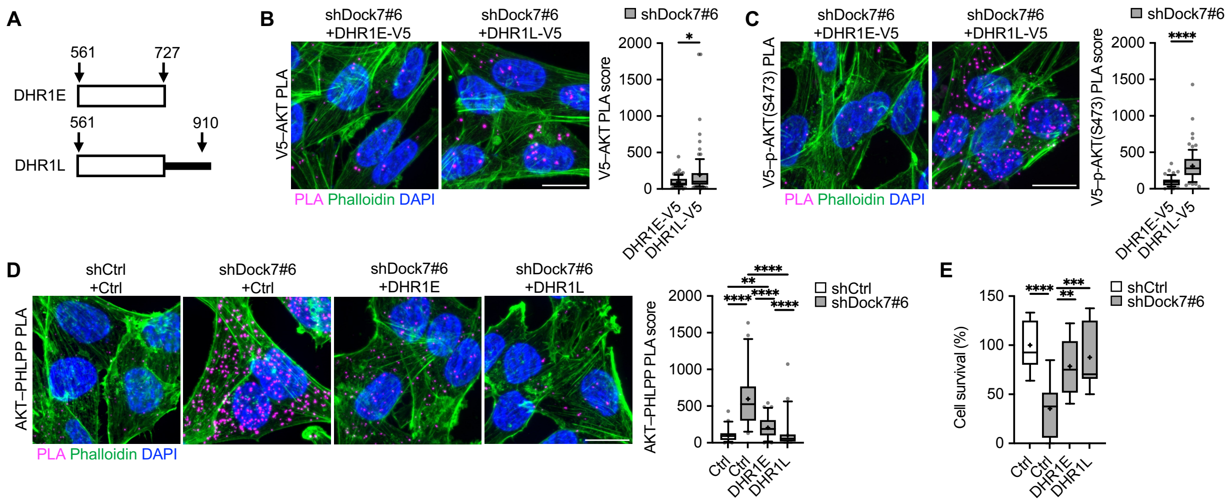

Figure S4. DHR1L is more effective than DHR1E at rescuing function following Dock7 knockdown. (**A**) Schematic of DHR1E and DHR1L constructs. (**B**) Cell survival of MDA-MB-231 cells following Dock7 knockdown and DHR1E and DHR1L overexpression after 2 days in serum-free media. (**C**-**E**) PLA image and score of (**C**) V5–AKT, (**D**) V5–p-AKT(S473), and (**E**) AKT–PHLPP interactions in MDA-MB-231 cells after overnight serum starvation following Dock7 knockdown and DHR1E and DHR1L overexpression. Data shown as median ± interquartile range (box), 5th–95th percentiles (whiskers), and mean (+). *P<0.05, **P<0.01, ***P<0.001 ****P<0.0001, ns = not significant. Scale bar = 15 μm.

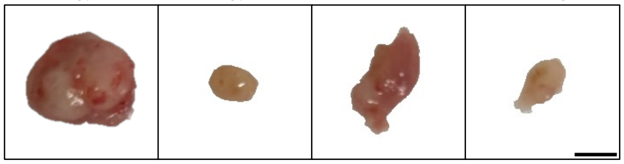

Figure S5. NSG xenograft tumor growth after 8 weeks. Representative images of NSG mouse tumor xenograft of MDA-MB-231 cells following Dock7 knockdown and DHR1L and DHR1L-C2M limit domain overexpression after 8 weeks. Scale bar = 5 mm.

Table S1. PLA antibodies.

Pair	Ms antibody	Rb antibody
Dock7–TSC1	Dock7 (1:100; Santa Cruz Biotechnology, sc-398888)	TSC1 (1:100, Cell Signaling Technology, 6935S)
Dock7–TSC2	Dock7 (1:100; Santa Cruz Biotechnology, sc-398888)	TSC2 (1:100; Cell Signaling Technology, 4308S)
Dock7–mTOR	Dock7 (1:100; Santa Cruz Biotechnology, sc-398888)	mTOR (1:100; Cell Signaling Technology, 2983S)
Dock7–Rheb	Dock7 (1:100; Santa Cruz Biotechnology, sc-398888)	Rheb (1:100, Cell Signaling Technology, 13879S)
Dock7–Raptor	Dock7 (1:100; Santa Cruz Biotechnology, sc-398888)	Raptor (1:100; Cell Signaling Technology, 2290S)
Dock7–Rictor	Dock7 (1:100; Santa Cruz Biotechnology, sc-398888)	Rictor (1:100; Cell Signaling Technology, 2114S),
Dock7–LAMP1	Dock7 (1:100; Santa Cruz Biotechnology, sc-398888)	LAMP1 (1:100, Cell Signaling Technology, 9091S)
Dock7–AKT	Dock7 (1:100; Santa Cruz Biotechnology, sc-398888)	AKT (1:100; Cell Signaling Technology, 9272S)
AKT–PHLPP	AKT (1:100, Cell signaling Technology, 2920S)	PHLPP (1:50, Proteintech, 22789-1-AP)
Dock7–p-AKT(S473)	p-AKT(S473) (1:50, Cell Signaling Technology, 4051S)	Dock7 (1:100; Abcam, ab118790)
p-AKT(S473)–p-TSC2(T1462)	p-AKT(S473) (1:50, Cell Signaling Technology, 4051S)	p-TSC2(T1462) (1:50, Cell Signaling Technology, 3617S)
V5–AKT	V5-tag (1:100, Cell Signaling Technology, 80076S)	AKT (1:100; Cell Signaling Technology, 9272S)
V5–p-AKT(S473)	p-AKT(S473) (1:50, Cell Signaling Technology, 4051S)	V5-tag (1:100, Cell Signaling Technology, 13202S)
Dock7–Cdc42	Dock7 (1:100; Santa Cruz Biotechnology, sc-398888)	Cdc42 (1:100, Cell Signaling Technology, 2466S)
V5–Cdc42	V5-tag (1:100, Cell Signaling Technology, 80076S)	Cdc42 (1:100, Cell Signaling Technology, 2466S)
