# Supplementary material for: A multiprotein signaling complex sustains AKT and mTOR/S6K activity necessary for the survival of cancer cells undergoing stress
Source: bioRxiv. 2024 Feb 27:2023.01.03.522657. Originally published 2023 Jan 3. Preprint. [Version 2] doi: 10.1101/2023.01.03.522657 (PMC9881951; doi:10.1101/2023.01.03.522657)
Supplement: Supplementary file 1 [file media-1.pdf]

Supplementary figures

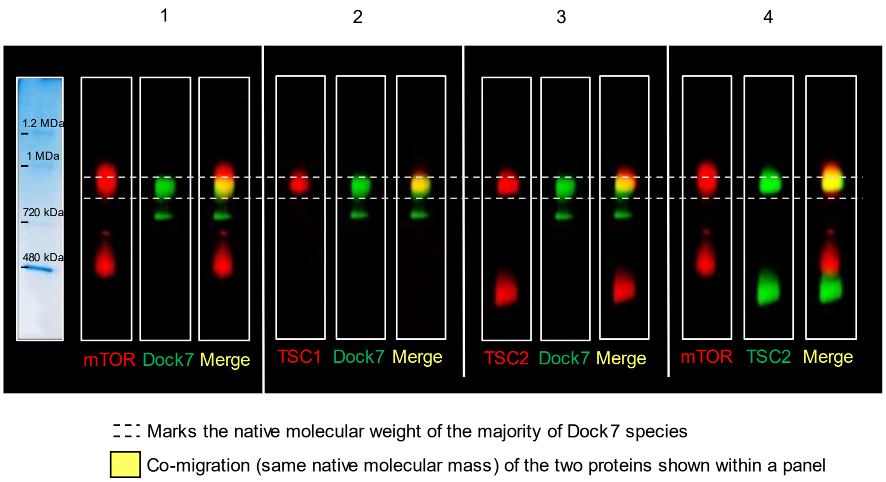

Supplemental Figure S1. Dock7 co-migrates with mTOR and its main negative regulator, the TSC1/2 complex. HEK-293 cells grown in complete medium were lysed and native complexes were separated using Blue Native-PAGE (BN-PAGE). Each lane was stained by the specified antibodies.

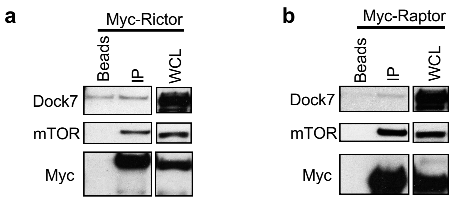

Supplemental Figure S2. Western blot of immunoprecipitated endogenous Dock7 and mTOR from HEK-293T cells transiently expressing (**a**) Myc-Rictor and (**b**) Myc-Raptor.

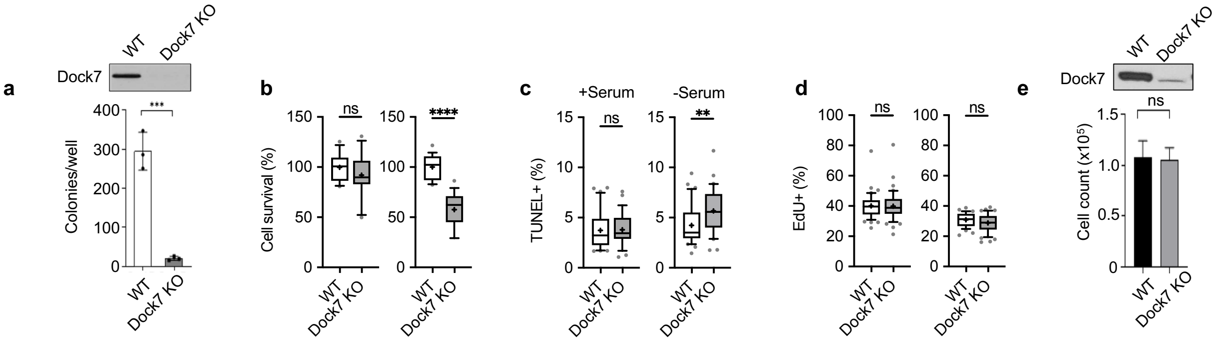

Supplemental Figure S3. HeLa Dock7 knock-out cell line. (**a**) HeLa WT and Dock7 KO cells were seeded in soft agar suspension, and colonies were counted two weeks later. (**b**) Cell survival assay of HeLa WT and Dock7 KO cells assessing cell count after 2 days in full-serum or serum-free media. (**c**) TUNEL assay of HeLa WT and Dock7 KO cells cultured in complete media or serum starved overnight. (**d**) EdU assay to determine cell proliferation of HeLa WT and Dock7 KO cells performed in complete media or after overnight serum starvation. (**e**) Dock7 heterozygous mice were crossed, and embryos were isolated at dpc13.5 to create Mouse Embryonic Fibroblasts (MEFs). Each MEF cell line was expanded and used for Western blot analysis to determine Dock7 protein expression. Dock7 KO and WT cells were then used for survival assays in serum free media.

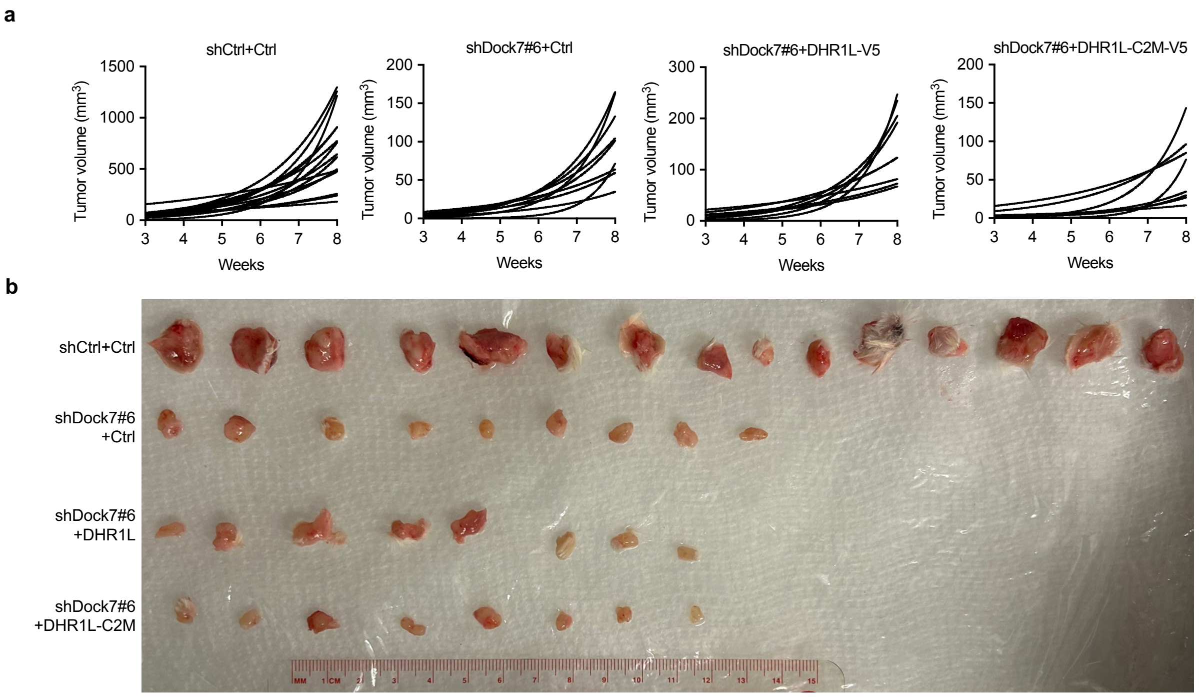

Supplemental Figure S4. NSG mouse tumor xenograft of MDA-MB-231 cells following Dock7 knockdown and DHR1L and DHR1L-C2M overexpression. (**a**) Tumor volume over 3-8 weeks post-injection was normalized to initial tumor volume at week 3, to assess the growth rate of each tumor. (**b**) Tumors harvested at week 8.

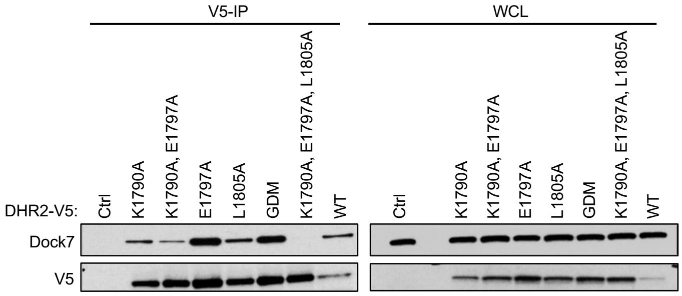

Supplemental Figure S5. Generation of a Dock7 DHR2 dimerization domain mutant, DHR2-DDM. Site-directed mutagenesis was used to mutate specific amino acids within the DHR2 domain of Dock7. Each mutant was semi-stably overexpressed in HEK-293T cells and immunoprecipitation studies were performed to isolate complexes between DHR2 and endogenous Dock7 protein (240KDa).
