# Supplementary material for: Dock7 regulates AKT and mTOR/S6K activity required for the transformed phenotypes and survival of cancer cells
Source: bioRxiv. 2023 Jan 3:2023.01.03.522657. Preprint. [Version 1] doi: 10.1101/2023.01.03.522657 (PMC9881951; doi:10.1101/2023.01.03.522657)
Supplement: Supplementary file 1 [file media-1.pdf]

Supplementary Figures

Supplemental Figure 1

A

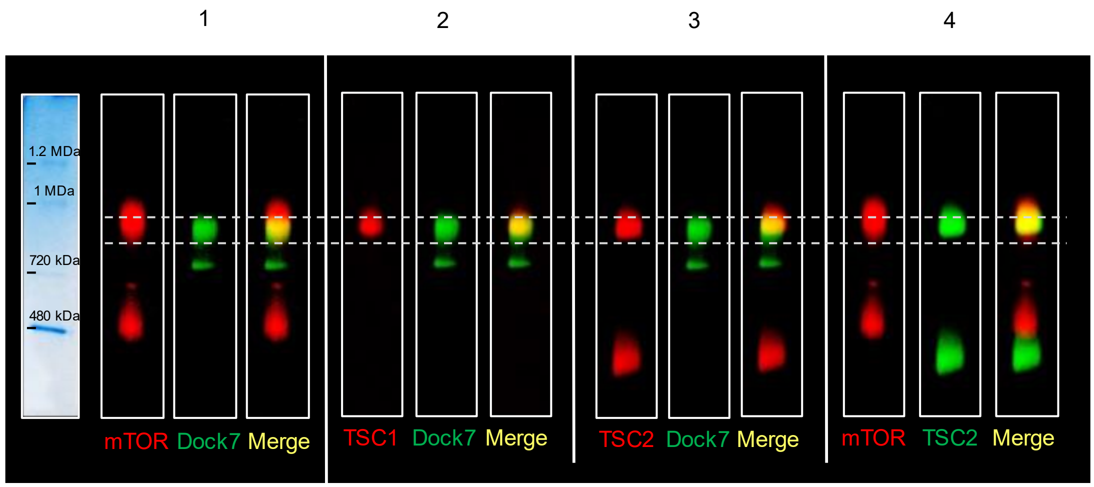

- - - Marks the native molecular weight of the majority of Dock7 species

Co-migration (same native molecular mass) of the two proteins shown within a panel

Supplemental Figure S1 Dock7 interacts with mTOR and its main negative regulator, the TSC complex. (**A**) HeLa cells growing in full serum were lysed and native complexes were separated using Blue Native-PAGE (BN-PAGE). Each lane was stained by the specified antibodies

Supplemental Figure 2

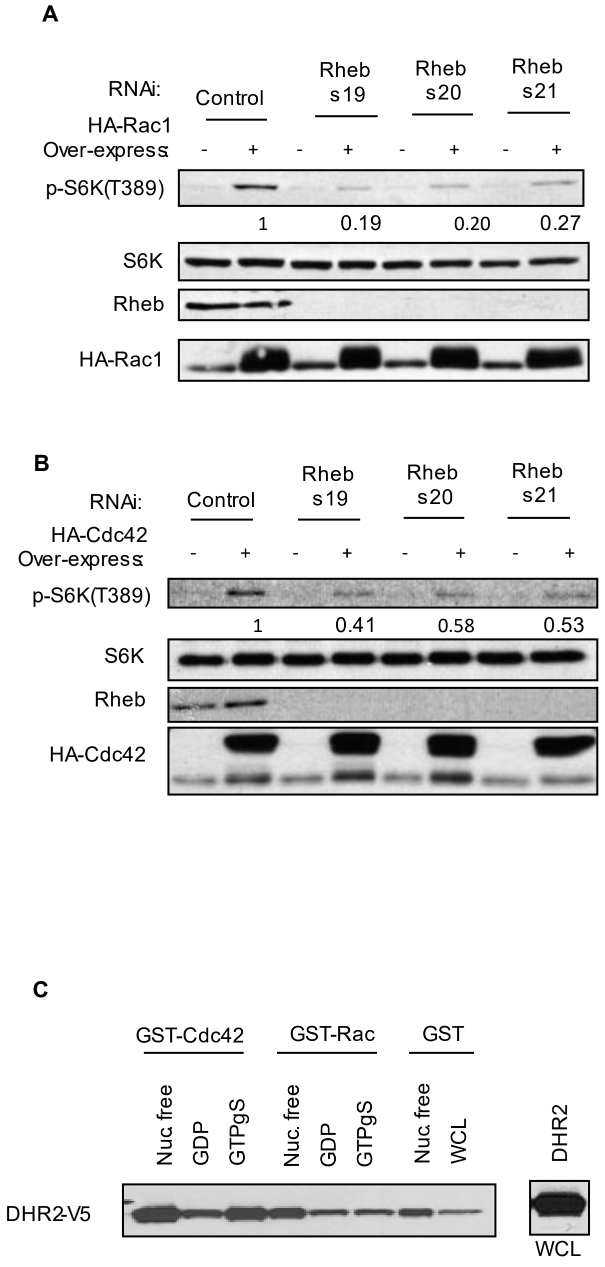

Supplemental Figure S2 Rac and Cdc42 require Rheb to stimulate mTORC/S6K activity. (**A** and **B**) Either Rac1 or Cdc42 was overexpressed while Rheb siRNAs were used to downregulate Rheb protein in WT HeLa cells. (**C**) Recombinant GST-tagged Cdc42 or Rac protein that was loaded with either GTPγs, GDP, or EDTA-treated was used to pull down DHR2-V5 from semi-stably expressing HEK293T cells. Blots were probed with V5 antibody to determine small GTPase-DHR2 binding. Numbers under western blots represent fold difference between phosphorylation states when when normalized to endogenous protein levels

Supplemental Figure 3

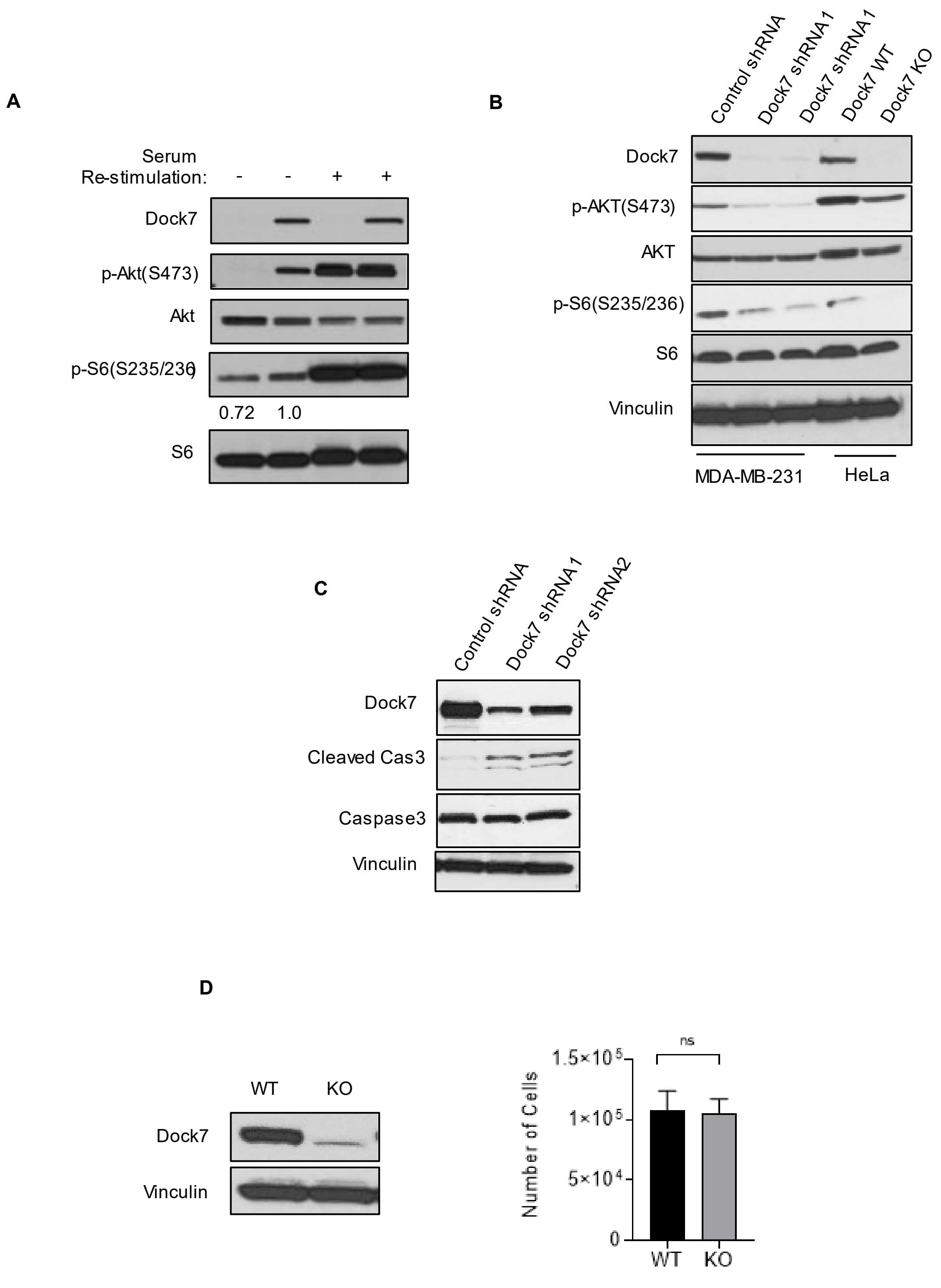

Supplemental Figure S3 Dock7 knock-out impairs transformative properties through increased apoptosis. (**A**) Crispr-Cas9 Dock7 KO and WT cells were seeded and allowed to

recover for a day before changing media to serum-free media for 24 h, serum restimulation was performed with dialyzed serum for 1 h prior collection and lysis. (**B**) Either MDA-MB-231 cells where Dock7 was knocked down using shRNA or Crispr-Cas9 Dock7 KO and WT HeLa cells were seeded in 100mm plates, allowed to recover for 20 h, and then grown at 1% O_2 for 24 h. Cells were then collected, and western blot analysis was performed. (**C**) Dock7 was knocked down from HeLa cells using shRNA, cells were selected with antibiotic, seeded, allowed to recover for 24 h, starved overnight, and then collected for Western blot analysis. (**D**) Dock7 heterozygous mice were crossed, and embryos were isolated at dpc13.5 to create Mouse Embryonic Fibroblast (MEF). Each MEF cell line was expanded and used western blot analysis to determine Dock7 protein expression. Dock7 KO and WT cells were then used to for survival assays.

Supplemental Figure 4

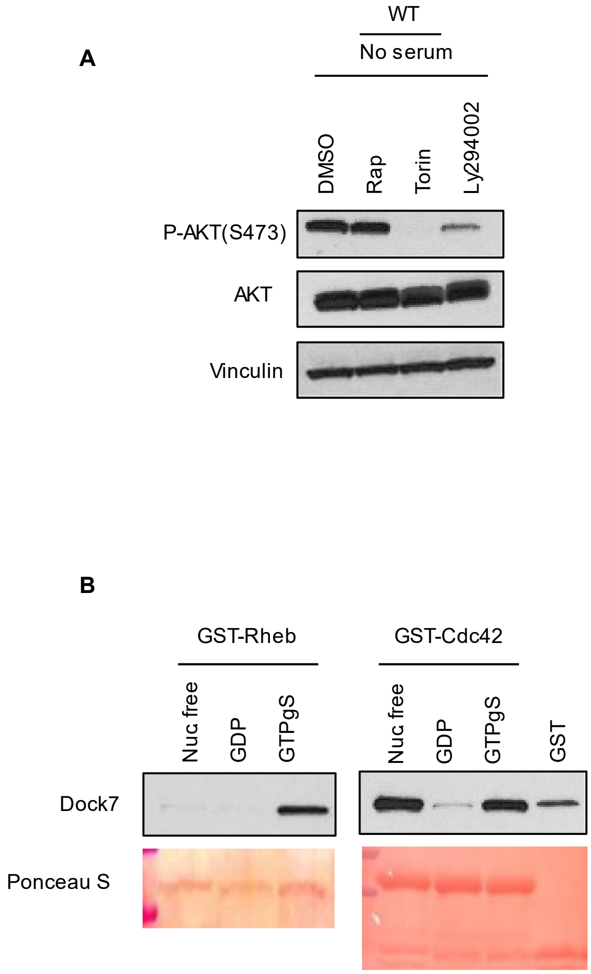

Supplemental Figure S4. Dock7-mediated AKT/mTOR activity in serum-deprived conditions is not fully dependent on PI3K but requires Rheb. (**A**) WT HeLa cells were seeded and allowed to recover for one day before media was changed to serum-free media containing either vehicle DMSO, Rapamycin (1 nM), Torin (100nM), or Ly294002 (50 µM). Cells were treated for 20 h, then collected, and lysed for Western blot analysis. (**B**) Recombinant GST-tagged Rheb protein that was loaded with either GTPγs, GDP, or EDTA-treated was used to pull down endogenous Dock7 from HEK293T cells. Blots were probed with anti-Dock7 antibody to determine small GTPase-Dock7 binding. Cdc42-GST was used as control.

Supplemental Figure 5

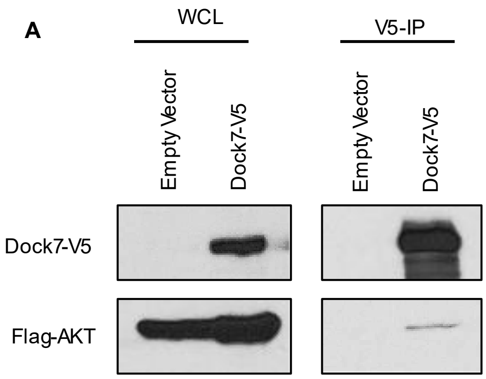

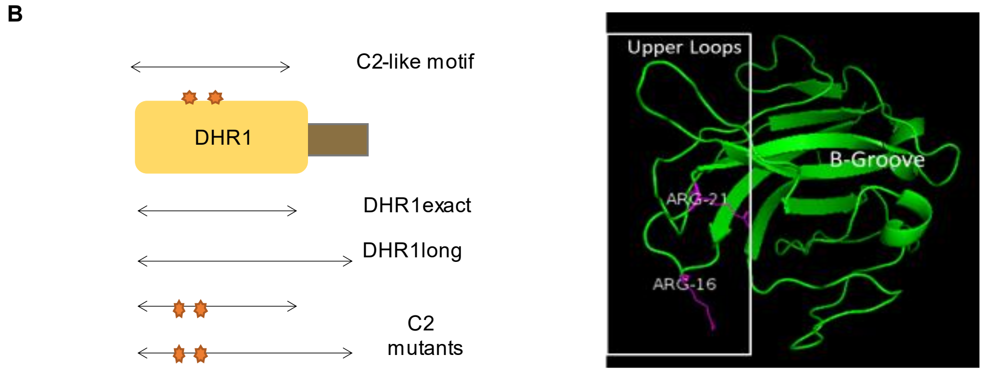

Supplemental Figure S5 Dock7 and AKT interact. (**A**) Flag-tagged AKT and Dock7-V5 were transiently overexpressed in HEK293T cells. Immunoprecipitation was performed using anti-V5 beads and complexes were resolved on an SDS-PAGE gel for Western analysis. (**B**) Depiction of the different DHR1 truncations and mutants created to test the functional role of DHR1 in AKT signaling, and Alphafold predicted structure for the DHR1 domain of Dock7
